# Photoinduced Low-Spin → High-Spin Mechanism of an Octahedral Fe(II) Complex Revealed by Synergistic Spin-Vibronic Dynamics

**DOI:** 10.1021/acs.inorgchem.1c01838

**Published:** 2021-09-09

**Authors:** Mátyás Pápai

**Affiliations:** Wigner Research Centre for Physics, P.O. Box 49, Budapest H-1525, Hungary

## Abstract

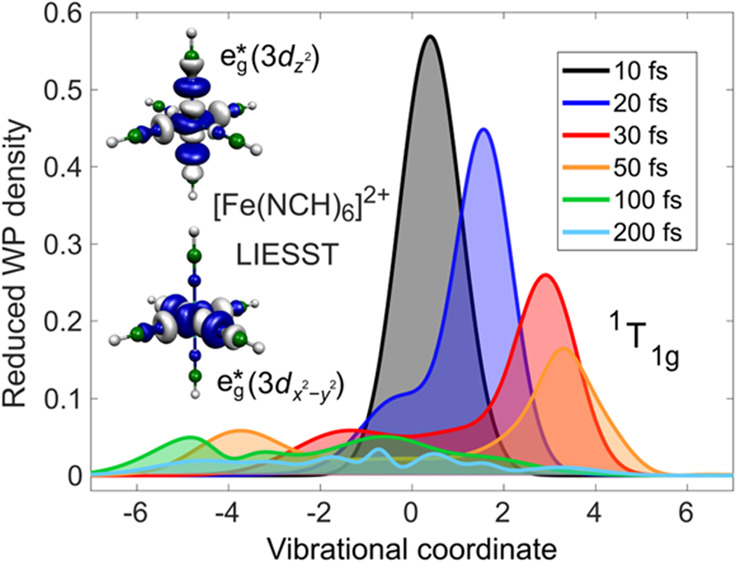

The Fe(II) low-spin (LS; ^1^A_1g_, t_2g_^6^e_g_^0^) → high-spin (HS; ^5^T_2g_, t_2g_^4^e_g_^2^) light-induced excited spin state trapping (LIESST) mechanism solely involving metal-centered states is revealed by synergistic spin-vibronic dynamics simulations. For the octahedral [Fe(NCH)_6_]^2+^ complex, we identify an initial ∼100 fs ^1^T_1g_ → ^3^T_2g_ intersystem crossing, controlled by vibronic coupling to antisymmetric Fe–N stretching motion. Subsequently, population branching into ^3^T_1g_, ^5^T_2g_ (HS), and ^1^A_1g_ (LS) is observed on a subpicosecond time scale, with the dynamics dominated by coherent Fe–N breathing wavepackets. These findings are consistent with ultrafast experiments, methodologically establish a new state of the art, and will give a strong impetus for further intriguing dynamical studies on LS → HS photoswitching.

## Abstract 2

The low-spin (LS) → high-spin (HS) light-induced excited spin-state trapping (LIESST) mechanism solely involving metal-centered states is revealed by synergestic spin-vibronic dynamics simulations. For the [Fe(NCH)_6_]^2+^ complex, we observe <100 fs ^1^T_1g_ → ^3^T_2g_ intersystem crossing and subsequent population branching into the ^3^T_1g_, ^5^T_2g_ (HS), and ^1^A_1g_ (LS) states. The intersystem crossing dynamics is found to be controlled by vibronic coupling to antisymmetric Fe−N stretching modes and coherent breathing wavepackets.

Photoinduced low-spin (LS) → high-spin (HS) transition in transition-metal complexes, known as light-induced excited spin state trapping (LIESST), has attracted great interest since its discovery in 1984.^1^ LIESST is an intriguing phenomenon both from the point of view of the fundamentals of excited-state processes and revolutionary applicational areas, such as molecular data storage.^2^ It is known that the LS-to-HS transition can also be triggered by varying the temperature, pressure, and magnetic field;^3^ in fact, the first discovery of these so-called spin-crossover (SCO) complexes is dated way back to 1931.^4^

In the past two decades, the LIESST mechanism has been a very “hot” research topic; this is motivated by the fact that the gained knowledge may allow control of excited-state pathways and thus can lead to the design of improved functional molecules and technologies. In the case of switchable hexacoordinated complexes with a Fe^II^N_6_ core, which by far dominate the known LIESST-exhibiting systems, irradiation of light converts the singlet ground state [^1^A_1g_(LS), t_2g_^6^e_g_^0^] into a quintet metastable state [^5^T_2g_(HS), t_2g_^4^e_g_^2^]; thus, a Δ*S* = 2 net change of the spin momentum occurs. The LIESST mechanism was first investigated in 1991 by Hauser^5^ for the 1-propyltetrazole [Fe(ptz)_6_](BF_4_)_2_ pseudooctahedral SCO Fe(II) complex, promoted to the lowest singlet metal-centered (MC) state. Utilizing UV/vis/near-IR absorption spectroscopy, he identified a triplet intermediate state, which at 10 K decays via branching into a quintet metastable state [^5^T_2g_(HS), 75%] and the ground state [^1^A_1g_(LS), 25%], occurring via intersystem crossing (ISC), as illustrated in Figure 1. More recently, for the Zn-doped crystal [Zn_0.9_Fe_0.1_(ptz)_6_](BF_4_)_2_, probed at 125 K by time-resolved absorption spectroscopy, Hauser and co-workers identified the upper triplet ^3^T_2g_ as the intermediate state, which is formed from ^1^T_1g_ in <150 fs and decays into the ^5^T_2g_ HS state in 1.2 ps.^6^ The HS state is metastable at low temperatures (*T* < 50 K), and its lifetime is determined by quantum-mechanical tunneling.

**Figure 1:**
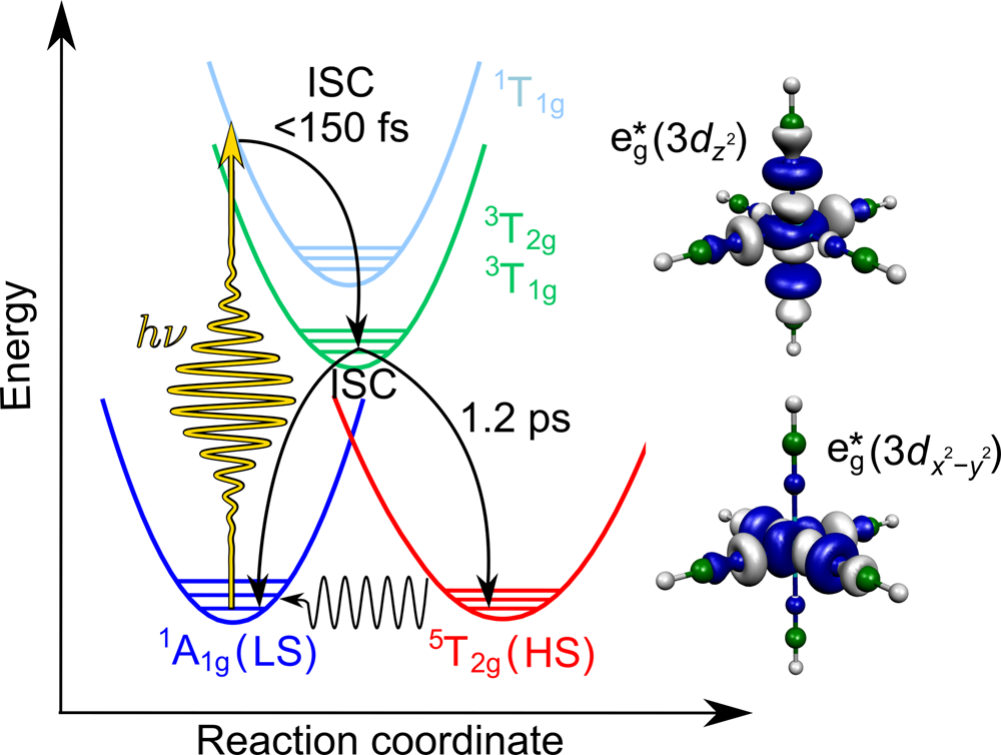
Schematic mechanistic picture of the LIESST effect in the case of excitation into the lowest ^1^MC (^1^T_1g_) state by 530 nm light. The wavy arrow illustrates the low-temperature HS → LS relaxation by tunneling. The excited-state lifetimes are taken for [Zn_0.9_Fe_0.1_(ptz)_6_](BF_4_)_2_ from ref (6). The electronic state labels are consistent with *O*_*h*_ point group symmetry. Also shown are the e_g_* antibonding orbitals of the complex investigated in the present work, [Fe(NCH)_6_]^2+^, which are populated in the excited states.

Despite these valuable mechanistic insights, several unknowns remain, in most cases, because of the ambiguities in the interpretation of the experimental data. An excellent example is the more complicated case of [Fe(bipy)_3_]^2+^ (bipy = 2,2′-bipyridine), a LS Fe(II) complex, which became the LS ↔ HS photoswitching prototype for time-resolved investigations. [Fe(bipy)_3_]^2+^ is converted to the HS state in <100 fs, initiated by irradiation into the optically active singlet metal-to-ligand charge-transfer (MLCT) band. This <100 fs time scale and the HS lifetime (ca. 650 ps in aqueous solution) are consistent for various experimental studies, but it is strongly debated how the HS state is populated.^7−9^ A final consensus is yet to be established; in order to achieve this goal, theory, which has the potential to complement and support time-resolved experiments, has a crucial role. However, the computational state of the art for light-induced singlet-to-quintet transitions has so far been limited to analysis of the static potential energy surfaces (PESs)^10^/couplings^11^ and estimation of the excited-state lifetimes by Fermi’s golden rule.^12,13^ Albeit often useful, these approaches face severe shortcomings when it comes to fast electronic relaxation such as LIESST, with a time scale comparable to that of nuclear motion. In fact, this nuclear–electronic coupling, leading to a dynamic mixing of the electronic states, is among the main sources for the complexity of the obtained time-resolved experimental data, which theory is intended to alleviate.

In this Communication, we present the first theoretical dynamics study on LS → HS photoswitching with excitation into ^1^MC states (^1^T_1g_, [Fe(ptz)_6_]^2+^ prototype; Figure 1). In order to achieve feasibility at a high computational level, we investigate the excited-state dynamics of [Fe(NCH)_6_]^2+^, a well-established model^14−17^ for metastable Fe(II)-based SCO systems. Its validity, with a special emphasis on LIESST, is discussed in the Supporting Information (SI). Employing a synergistic spin-vibronic approach,^18,19^ we achieve an invaluable mechanistic understanding, whose adequacy is assessed and confirmed by its consistency with the above-discussed experiments.

Herein, we employ a synergistic approach, which exploits the complementary character of trajectory surface hopping (TSH; full dimensionality) and quantum dynamics (QD; fully quantum description). We utilize full-dimensional TSH on potentials computed on-the-fly to select the dominant nuclear degrees of freedom and QD on high-level ab initio (CASPT2) precomputed surfaces along the selected modes for the accurate simulation of LIESST excited-state dynamics. A brief methodological description is provided in the Computational Methods section, with details given in the SI.

Figure 2 presents the normal-mode activity of the vibrational motion, obtained from the excited-state TSH trajectories. As is clear from the figure, three modes, ν_13_, ν_14_, and ν_15_, dominate the excited-state nuclear motion. As shown in Figure 3, all three modes have Fe–N stretching character, but while the 2-fold-degenerate ν_13_ and ν_14_ modes are antisymmetric, ν_15_ is a totally symmetric (breathing) mode. These three Fe–N stretching modes are in excellent agreement with the natural choice of coordinates upon the occupation of e_g_* antibonding orbitals, shown in Figure 1, in MC excited states.

**Figure 2:**
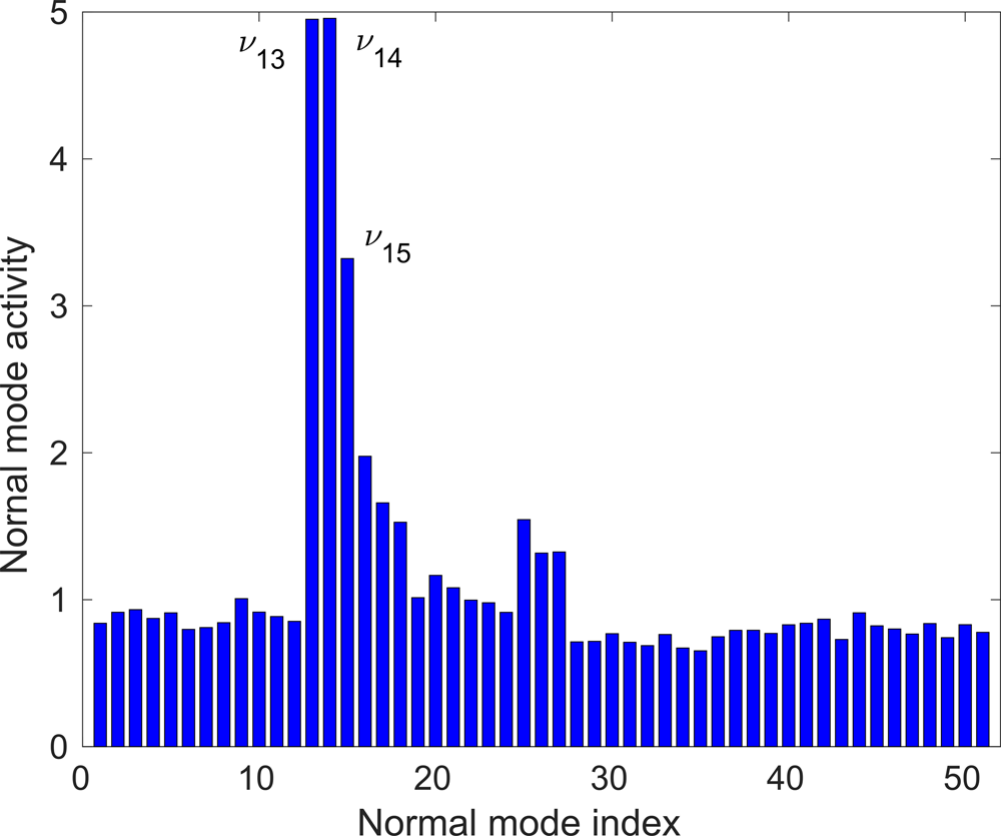
Dynamic normal-mode activity, as determined from the standard deviation of nuclear displacements, projected onto the ground-state normal modes. This was obtained from the singlet–triplet TSH simulations. The labels of the three dominant normal modes, ν_13_, ν_14_, and ν_15_, are depicted. As illustrated in Figure 3, the character of these three modes is Fe–N stretching.

**Figure 3:**
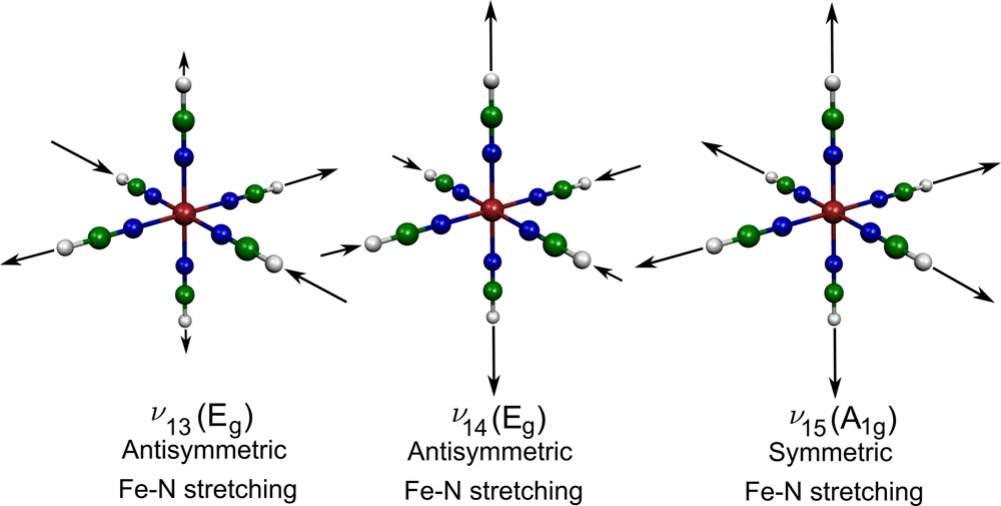
Illustration of the three dominant modes of [Fe(NCH)_6_]^2+^, ν_13_ (antisymmetric Fe–N stretching), ν_14_ (antisymmetric Fe–N stretching), and ν_15_ (symmetric Fe–N stretching), identified by the TSH simulations. Also shown are the *O*_*h*_ symmetry labels of the modes.

Figure 4 shows the PESs along modes ν_15_ and ν_14_; the PESs along ν_13_ are shown in Figure S7. While the ν_15_ breathing mode is crucial to connect the LS (^1^A_1g_) and HS (^5^T_2g_) states (Figure 4a), it maintains the octahedral symmetry and does not allow the different singlet and triplet MC components to cross. The reason for this is that, in the singlet and triplet excited states, a single e_g_* orbital is populated, which activates antisymmetric Fe–N stretching modes, while the ν_15_ breathing mode is totally symmetric. Indeed, as seen in Figure 4b, the excited-state potentials split along ν_14_, allowing the possibility of singlet–triplet ISC and triplet internal conversion (IC) via the intersection of the corresponding MC PESs.

**Figure 4:**
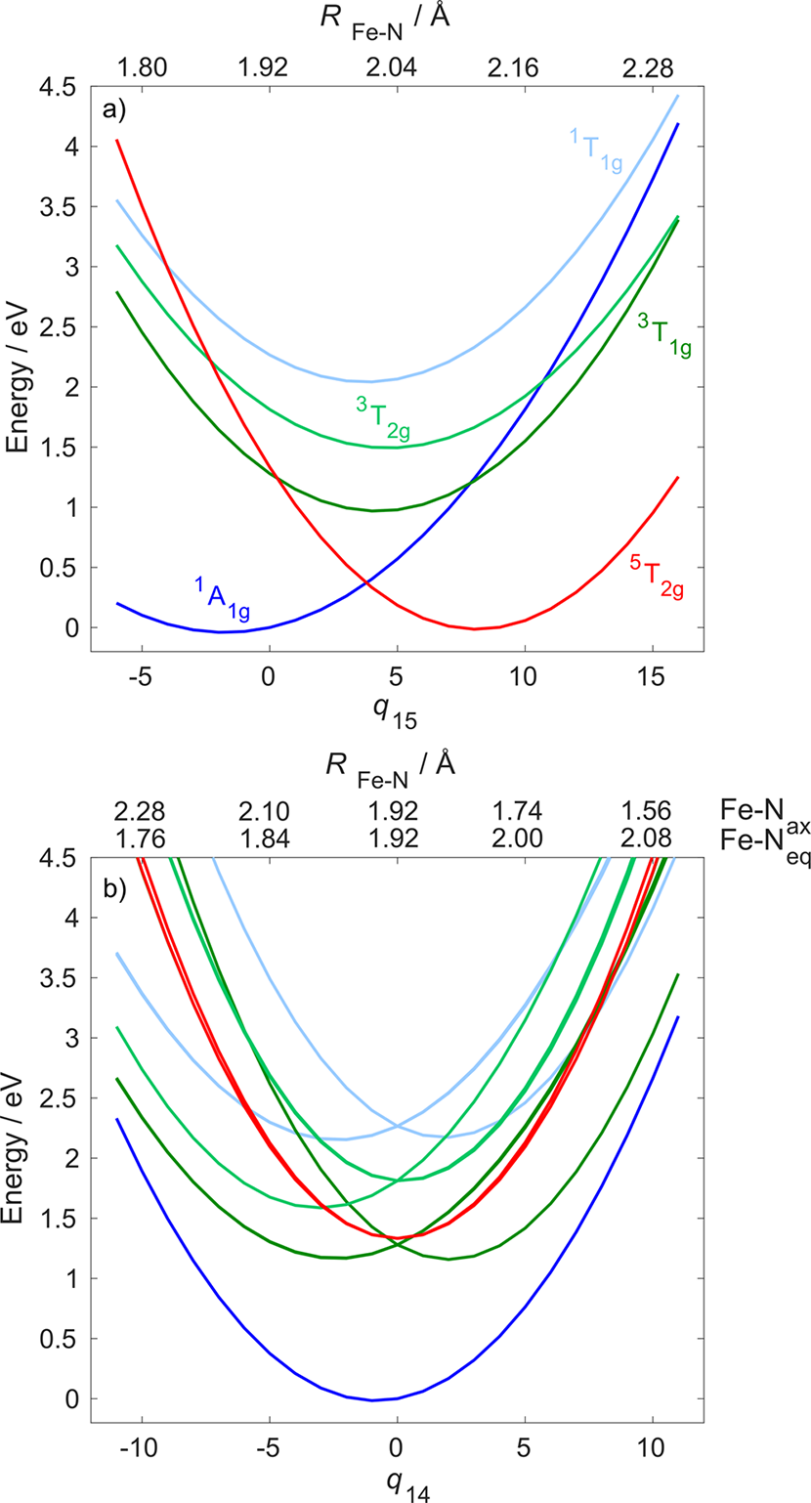
One-dimensional cuts of the diabatic CASPT2 PESs along (a) ν_15_ and (b) ν_14_. Nuclear displacements are given in dimensionless mass-frequency-scaled normal coordinates and Fe–N distances (Å).

In Figure 5, we present the electronic population dynamics, as obtained from the three-dimensional QD simulation utilizing the selected modes and all relevant singlet–triplet–quintet states. As shown in the figure, the ^1^T_1g_ population decay has two components, characterized by the 39 and 168 fs exponential time constants. These time scales are in excellent agreement with the experimentally observed <150 fs ISC.^6^ As proposed by Marino et al.,^6^ the ^1^T_1g_ states indeed deactivate via the ^3^T_2g_ states. Subsequently, the excited-state population flows on a subpicosecond time scale to ^3^T_1g_ by triplet IC and to the ^5^T_2g_ manifold via triplet–quintet ISC; as a minor component, the ground state ^1^A_1g_ is recovered. The ^3^T_1g_ population is stable on the 1 ps time duration of the simulation, which is consistent with its 39 ps^6^ lifetime detected experimentally.^20^ Crucially, the simulated dynamics captures all important aspects of the LIESST mechanism revealed by experiments: very fast singlet–triplet ISC, the role of ^3^T_2g_ states as intermediates, and branching into the HS/LS states. This consistency supports the presented results, which for certain aspects, such as the singlet–triplet ISC, reach even a quantitative agreement with experiment.^6^

**Figure 5:**
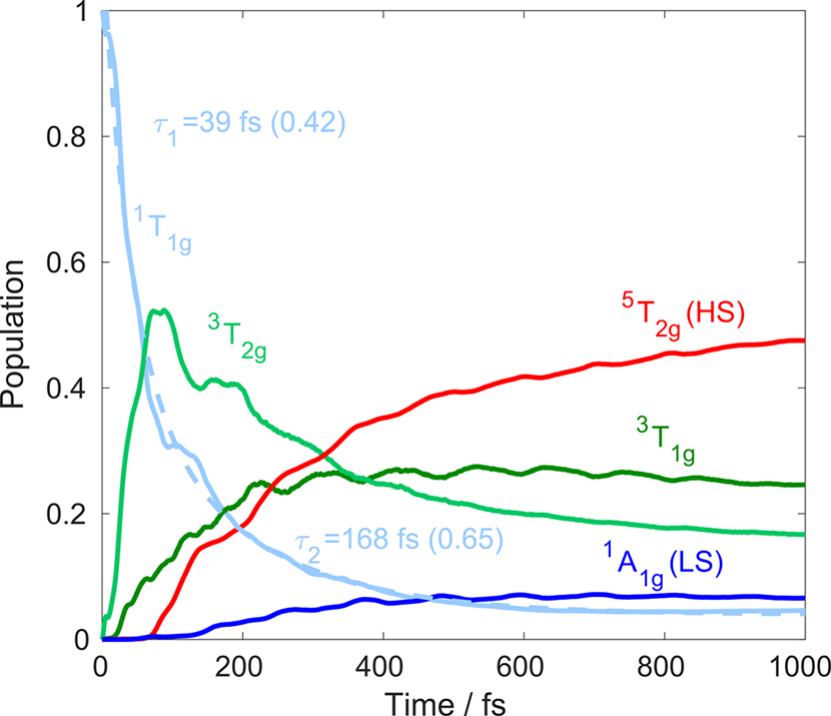
Simulated population dynamics upon excitation into the ^1^T_1g_ manifold. The ^1^T_1g_ population curve (light blue) is fitted with a biexponential functional (dashed line); the numbers in parentheses denote the coefficients of the two exponential components.

Finally, we reveal fine details of the LIESST mechanism based on the time evolution of excited-state nuclear wavepackets. We now analyze the singlet–triplet ISC along ν_14_, which is the natural choice for the involved ^1^T_1g_/^3^T_2g_ states (occupation of a single e_g_* orbital). Figure 6a shows snapshots of the excited-state singlet wavepacket along ν_14_ for the initial 200 fs. The ^1^T_1g_ wavepacket immediately starts to propagate away from *q*_14_ = 0, entering the ^1^T_1g_/^3^T_2g_ crossing region. This, combined with a sizable spin–orbit coupling (∼175 and 250 cm^–1^), allows efficient ISC to the ^3^T_2g_ manifold, which is indeed what is observed in Figure 5. On the 100–200 fs time scale, the wavepacket becomes more diffuse, which is the reason why the ISC is slower for >100 fs. These results highlight the importance of vibronic motion, as confirmed by the discrepancy between the 36 ps ^1^T_1g_ → ^3^T_2g_ ISC time constant of ref (13) for [Fe(mtz)_6_]^2+^, based on Fermi’s golden rule, and the <150 fs experimental^6^ and simulated 39/168 fs values obtained in this work.

**Figure 6:**
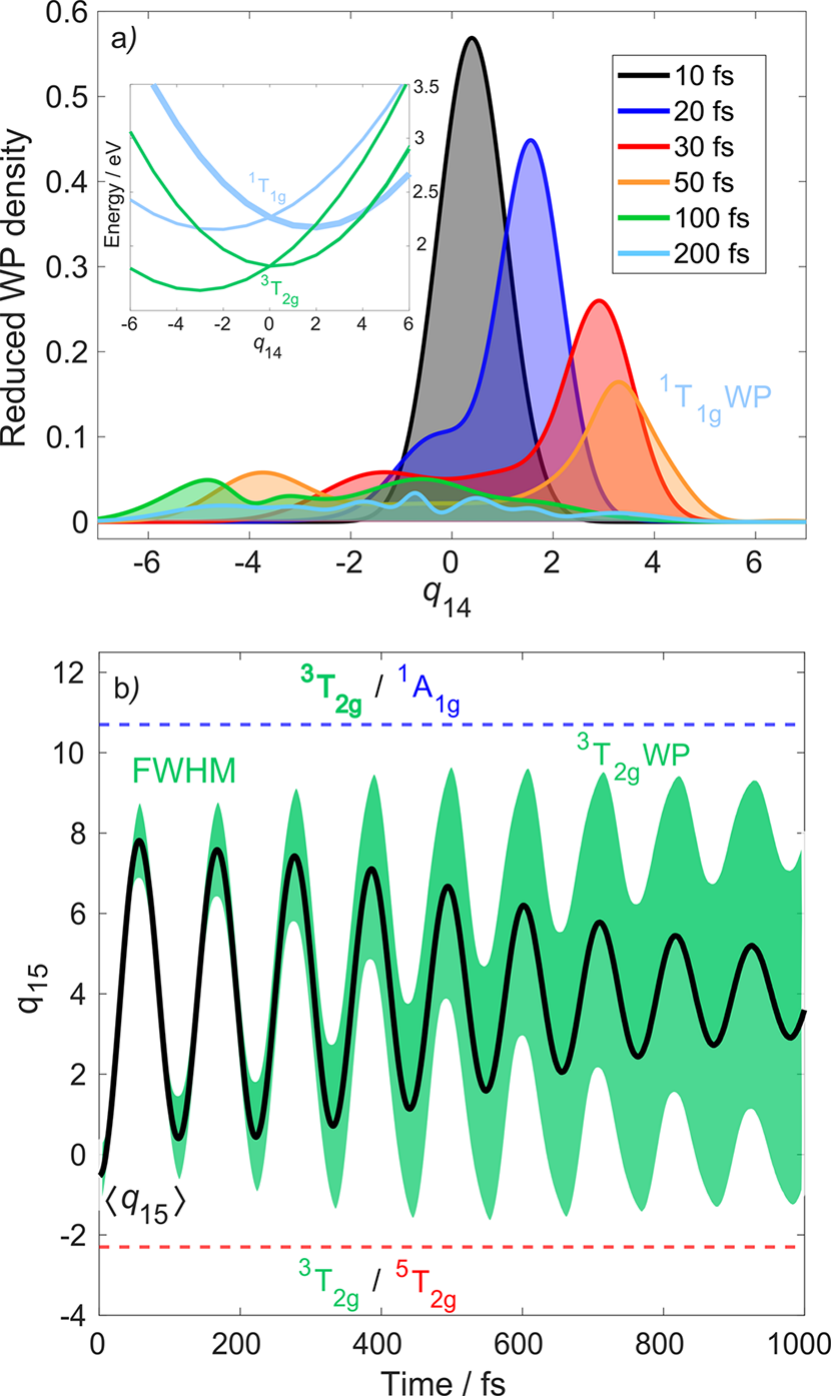
(a) Wavepacket (WP) dynamics along ν_14_ (^1^T_1g_ WP). The inset shows the relevant excited-state potentials (the ^1^T_1g_ component, from which the dynamics is initiated, is displayed using a thick light-blue line). (b) WP dynamics along ν_15_ (^3^T_2g_ WP), illustrated by the wavepacket centroid (black) and Gaussian width (green; fwhm). Also shown are the positions of the relevant intersections (^3^T_2g_/^5^T_2g_ and ^3^T_2g_/^1^A_1g_) along ν_15_ (dashed lines).

In Figure 6b, we analyze the wavepacket motion in the intermediate ^3^T_2g_ state along ν_15_. Clearly, a coherent wavepacket oscillation is induced, with a period of ∼110 fs. The wavepacket dephases and spreads, and, importantly, the Gaussian full width at half-maximum (fwhm, green) does not reach the crossings with the ^5^T_2g_ and ^1^A_1g_ states (dashed lines in Figure 6b). This is to say that the ^3^T_2g_ wavepacket only slightly leaks into the crossing region, which is the reason why the quintet population rises relatively slowly (in [Fe(bipy)_3_]^2+^, the HS state is populated in <100 fs^7,8^). Although the wavepacket gains more access to the ^3^T_2g_/^5^T_2g_ intersection along ν_14_, this channel does not allow significant net population flow to the ^5^T_2g_ states. This is due to very efficient ^5^T_2g_ → ^3^T_1g_ deactivation, favored by the energetics and easy access to the crossing region (located just at the ^5^T_2g_ minimum) along ν_14_, as is clear from Figure 4b.

In this Communication, we revealed the Fe(II) LIESST mechanism of Fe[(NCH)_6_]^2+^ using synergistic spin-vibronic dynamics simulations. The obtained mechanistic picture is consistent with the experimental findings and includes assignment of the principal intermediate ^3^T_2g_, whose dynamics are controlled by the key antisymmetric (ν_14_) and symmetric (ν_15_) Fe–N stretching vibrations. Importantly, the present work establishes a new theoretical state of the art for LS → HS photoswitching and will certainly motivate intriguing dynamical studies, both experimental and theoretical ones.

**Computational Methods**. The TSH simulations are based on Tully’s fewest switches,^21^ a three-step propagator technique^22^ (with transformations between the adiabatic–spin diabatic and diagonal representations), and local diabatization.^23^ The QD simulations are based on a diabatic vibronic-coupling^24,25^/spin-vibronic^26−29^ Hamiltonian and the multiconfigurational time-dependent Hartree (MCTDH) method.^30^ Our methodology uses the following implementations: TSH, *SHARC2.1*(31) interfaced to *ORCA4.2*(32,33) utilizing time-dependent density functional theory (TD-DFT; B3LYP*,^34,35^ singlet–triplet states including spin–orbit coupling); QD, Heidelberg *MCTDH8.4*;^30^ CASPT2 (12 electrons/14 orbitals: five–five Fe 3d/4d-based, two-σ_Fe–N_ bonding and a pair of Fe 3s/4s correlating active orbitals), *OpenMolcas20.10*.^36^ Further details of the utilized methodology are described in the SI.

## Supporting Information Available

Validity of the [Fe(NCH)_6_]^2+^ model, normal-mode frequencies and characters, methodological and computational details of the TSH and QD simulations, and optimized ground-state geometries (PDF)
