# Supplementary material for: Photoinduced Low-Spin → High-Spin Mechanism of an Octahedral Fe(II) Complex Revealed by Synergistic Spin-Vibronic Dynamics
Source: Inorg Chem. 2021 Sep 9;60(18):13950–4. doi: 10.1021/acs.inorgchem.1c01838 (PMC8456406; doi:10.1021/acs.inorgchem.1c01838)
Supplement: Supplementary file 1 — ic1c01838_si_001.pdf [file ic1c01838_si_001.pdf]

Supporting Information for "Photoinduced Low-Spin→High-Spin Mechanism of an Octahedral Fe(II) Complex Revealed by Synergistic Spin-Vibronic Dynamics"

Mátyás Pápai*

Wigner Research Centre for Physics, P.O. Box 49, H-1525 Budapest, Hungary

Email: papai.matyas@wigner.hu

Contents

S1 The $[Fe(NCH)_6]^{2+}$ LIESST Model **S2**

S2 TSH Simulations **S2**

S3 Normal Mode Analysis **S9**

S4 QD Simulations **S9**

S5 DFT-Optimized Ground-State Geometries **S26**

S1 The $[Fe(NCH)_6]^{2+}$ LIESST Model

In this work, we simulate the Fe(II) light-induced spin-state trapping (LIESST) dynamics, in the case of optical $d \rightarrow d$ excitation, using the $[Fe(NCH)_6]^{2+}$ model complex. Experimentally, the complex investigated was $[Fe(ptz)_6](BF_4)_2$ (ptz = 1-propyltetrazole), which is in fact the compound on which the LIESST mechanism was first studied.[1] $[Fe(NCH)_6]^{2+}$ is a well-established model for Fe(II) spin crossover (SCO), with the main focus on the computation of the high-spin (HS)-low spin (LS) energy gap.[2–5] However, the application of the $[Fe(NCH)_6]^{2+}$ model for LIESST requires further explanation, which we present below.

First, the LIESST mechanism of $[Fe(ptz)_6](BF_4)_2$, in the case when the dynamics is initiated by optical $d \rightarrow d$ excitation, exclusively involves metal-centered (MC) states. Thus, simplification of the ligands is justified, in contrast to cases involving metal-to-ligand charge transfer (MLCT) states. Second, $[Fe(ptz)_6]^{2+}$ and $[Fe(NCH)_6]^{2+}$ have the same Fe(II)N_6 core, with similar chemical environment; indeed, the multiconfigurational second-order perturbation theory (CASPT2) PESs of $[Fe(NCH)_6]^{2+}$ along the symmetric Fe-N stretching mode (ν_{15}, Figure 4a) are in very good agreement with those with the ligands 1-*H*-tetrazole[6] (tz) and 1-methyltetrazole[7] (mtz), which are much closer to ptz; this further supports the adequacy of the $[Fe(NCH)_6]^{2+}$ model. Furthermore, our CASPT2-calculated HS-LS energy gap of 210 cm^{-1} (26.1 meV) is in excellent agreement with SCO behaviour. The adequacy of the $[Fe(NCH)_6]^{2+}$ LIESST model is further confirmed by the very decent overall agreement between our CASPT2-based QD results and experiments on $[Fe(ptz)_6](BF_4)_2$.[1,8] As a final note, it is expected that the $[Fe(NCH)_6]^{2+}$ model would break down in cases for which the ligands play a decisive role such as MLCT excitation and energy transfer via ligand modes.

S2 TSH Simulations

As both trajectory surface hopping (TSH) and quantum dynamics (QD) employing a spin-vibronic Hamiltonian are known and well-documented methods, we here only briefly describe them. For further details, the reader is referred to the literature.[9–15]

In TSH, nuclear motion is treated classically by solving the equations of motion given by Newton's second law. The nuclear motion is dictated by the forces acting on the potential energy surface (PES); in most cases as also in this work, calculated on-the-fly within the Born-Oppenheimer approximation by a quantum chemistry program. Importantly, TSH is not restricted by the number of nuclear degrees of freedom (DoF), as the nuclear motion is classical, thus the computational bottleneck is solely determined by the electronic structure. However, this also means that quantum effects are not accounted for. Nevertheless, certain quantum aspects, such as electronic transitions and decoherence can be approximately taken into account. In TSH, electronic transitions are approximated by stochastic hops, whose probability is proportional to the nonadiabatic coupling. The electronic coefficients are propagated according to the electronic Schrödinger equation:

TSH is thus classified as a semi-classical molecular dynamics method.

TSH simulations were carried out using the SHARC2.1 code.[11] The classical equations of motion were integrated by a Velocity Verlet algorithm. A time step of 0.5 fs was used for the nuclear propagation. Surface-hopping probabilities were calculated[16] by a method based on Tully's fewest switches[17] and local diabatization (electronic propagation by wave function overlaps).[18] For the electronic propagation, we applied a time step of 0.005 fs. We used the correction scheme of Granucci *et al.*,[19] based on the overlap of frozen Gaussian wavepackets, to approximately account for electronic decoherence.

The electronic structure calculations for the TSH dynamics were carried out using ORCA4.2,[20,21] interfaced to SHARC. The present work focuses on the simulation of LIESST, for which intersystem crossing (ISC) has a central importance. The TSH dynamics thus has to include spin-orbit coupling (SOC). Therefore, we employ the diagonal electronic representation, which is obtained by diagonalization of the electronic Hamiltonian in the adiabatic basis, in which SOCs appear as off-diagonal elements. As a compromise between accuracy and computational cost, we include the forces only for those states in the transformation into the diagonal representation, whose potential energy is less than 0.5 eV, relative to the classically occupied active state.

For the studied $[Fe(NCH)_6]^{2+}$ complex, computational feasibility of on-the-fly TSH does not allow to go beyond the quantum chemical level of time-dependent density functional theory (TD-DFT). We employ the (TD-)B3LYP* exchange-correlation functional,[22,23] which, for Fe complexes. was found to consistently yield accurate excited-state energetics.[24–27] We use the TZVP basis set for all atoms. For TD-DFT, we utilize the Tamm-Dancoff approximation (TDA).[28] Two-electron integrals were approximated by the resolution of identity (RI-J) and chain of spheres (COSX) methods.[29] SOC for TD-DFT was calculated using the approach of Izsák and Neese *et al.* based on second-quantised spin operators and SOC integrals written in a spherical basis.[30] In these calculations we applied a mean-field/effective potential SOC operator[31] and the zeroth-order regular approximation (ZORA).[32,33]

From an electronic structure point of view, we face a methodological problem with TD-DFT. TD-DFT for excited states is based on linear response, which, utilizing a singlet reference (which is used for singlet and triplet excited-states), cannot access quintet states, as this would require double excitations. Although quintet states can be calculated using a quintet reference, this is not the case for triplet-quintet SOCs (the triplet states are obtained from a singlet-reference TD-DFT calculation). Therefore, our TSH model only includes singlet and triplet states: 4 singlets (the ground state plus 3 excited states) and 18 triplets (6 excited states, each possessing 3 spin components). These on-the-fly TD-DFT PESs (including SOC) allow to identify the most important nuclear motion in the singlet and triplet states. Although triplet-quintet ISC could not be taken into account, our methodology does allow identification of the dominant nuclear DoF responsible for accessing triplet-quintet crossing regions. We here emphasize that our intention with TSH is to identify the dominant excited-state nuclear motion for QD, not to be able to simulate the full dynamics.

100 initial conditions (nuclear geometries and velocities) were sampled from a Wigner

distribution based on the ground-state normal modes of $[Fe(NCH)_6]^{2+}$ calculated at the B3LYP*/TZVP level, whose frequencies and characters are given in Table S1. From these 100 initial conditions, 500 fs long trajectories were run starting from the S_4 adiabatic state (the highest-lying adiabatic singlet spin-free excited state, out of the 3 calculated). This technique corresponds to projection of the ground-state nuclear distribution into the $^1T_{1g}$ manifold.

Table S1: DFT-caculated ground-state normal modes of $[Fe(NCH)_6]^{2+}$. The B3LYP* and BP86 normal modes are utilized in the TSH and QD simulations, respectively. Obtained by using the TZVP basis set for all atoms. Frequencies are given in cm^{-1}. The mode labels are based on the B3LYP*-calculated frequency ordering (this mode notation is used in the main article as well).

Mode	Symmetry (O_h)	B3LYP*	BP86	Character
ν_1	t_{2u}	87.1	88.8	Rocking, bending (Fe-N-C)
ν_2	t_{2u}	87.1	88.8	Rocking, bending (Fe-N-C)
ν_3	t_{2u}	87.1	88.8	Rocking, bending (Fe-N-C)
ν_4	t_{2g}	106.7	108.8	Rocking, bending (Fe-N-C)
ν_5	t_{2g}	106.7	108.8	Rocking, bending (Fe-N-C)
ν_6	t_{2g}	106.7	108.8	Rocking, bending (Fe-N-C)
ν_7	t_{1u}	125.0	128.1	Rocking, bending (Fe-N-C)
ν_8	t_{1u}	125.0	128.1	Rocking, bending (Fe-N-C)
ν_9	t_{1u}	125.0	128.1	Rocking, bending (Fe-N-C)
ν_{10}	t_{1g}	248.6	261.7	Rocking, bending (Fe-N-C)
ν_{11}	t_{1g}	248.6	261.7	Rocking, bending (Fe-N-C)
ν_{12}	t_{1g}	248.6	261.7	Rocking, bending (Fe-N-C)
$\boldsymbol{\nu_{13}}$	e_g	299.1	331.9	Antisymmetric stretching (Fe-N)
$\boldsymbol{\nu_{14}}$	e_g	299.1	331.9	Antisymmetric stretching (Fe-N)
$\boldsymbol{\nu_{15}}$	a_{1g}	318.4	356.0	Symmetric stretching (Fe-N)
ν_{16}	t_{1u}	334.3	355.6	Fe off-center movement, bending (Fe-N-C)
ν_{17}	t_{1u}	334.3	355.6	Fe off-center movement, bending (Fe-N-C)
ν_{18}	t_{1u}	334.3	355.6	Fe off-center movement, bending (Fe-N-C)
ν_{19}	t_{2u}	361.1	383.4	Twisting (Fe-N-C, N-Fe-N)
ν_{20}	t_{2u}	361.1	383.4	Twisting (Fe-N-C, N-Fe-N)
ν_{21}	t_{2u}	361.1	383.4	Twisting (Fe-N-C, N-Fe-N)
ν_{22}	t_{2g}	376.0	395.9	Bending (Fe-N-C, N-Fe-N)
ν_{23}	t_{2g}	376.0	395.9	Bending (Fe-N-C, N-Fe-N)
ν_{24}	t_{2g}	376.0	395.9	Bending (Fe-N-C, N-Fe-N)
ν_{25}	t_{1u}	464.8	497.3	Bending (Fe-N-C, N-Fe-N)
ν_{26}	t_{1u}	464.8	497.3	Bending (Fe-N-C, N-Fe-N)
ν_{27}	t_{1u}	464.8	497.3	Bending (Fe-N-C, N-Fe-N)
ν_{28}	t_{1g}	786.0	731.1	Bending (N-C-H)

ν_{29}	t_{1g}	786.0	731.1	Bending (N-C-H)
ν_{30}	t_{1g}	786.0	731.1	Bending (N-C-H)
ν_{31}	t_{2u}	787.0	731.8	Bending (N-C-H)
ν_{32}	t_{2u}	787.0	731.8	Bending (N-C-H)
ν_{33}	t_{2u}	787.0	731.8	Bending (N-C-H)
ν_{34}	t_{1u}	787.7	733.0	Bending (N-C-H)
ν_{35}	t_{1u}	787.7	733.0	Bending (N-C-H)
ν_{36}	t_{1u}	787.7	733.0	Bending (N-C-H)
ν_{37}	t_{2g}	788.9	734.1	Bending (N-C-H)
ν_{38}	t_{2g}	788.9	734.1	Bending (N-C-H)
ν_{39}	t_{2g}	788.9	734.1	Bending (N-C-H)
ν_{40}	e_{g}	2238.4	2168.2	Antisymmetric stretching (N-C)
ν_{41}	e_{g}	2238.4	2168.2	Antisymmetric stretching (N-C)
ν_{42}	t_{1u}	2239.1	2168.0	Antisymmetric stretching (N-C)
ν_{43}	t_{1u}	2239.1	2168.0	Antisymmetric stretching (N-C)
ν_{44}	t_{1u}	2239.1	2168.0	Antisymmetric stretching (N-C)
ν_{45}	a_{1g}	2249.8	2189.8	Symmetric stretching (N-C)
ν_{46}	e_{g}	3373.1	3324.2	Antisymmetric stretching (C-H)
ν_{47}	e_{g}	3373.1	3324.2	Antisymmetric stretching (C-H)
ν_{48}	t_{1u}	3373.2	3324.2	Antisymmetric stretching (C-H)
ν_{49}	t_{1u}	3373.2	3324.3	Antisymmetric stretching (C-H)
ν_{50}	t_{1u}	3373.2	3324.3	Antisymmetric stretching (C-H)
ν_{51}	a_{1g}	3378.7	3331.6	Symmetric stretching (C-H)

Figure S1 presents the electronic population dynamics with all three singlet excited states ($^1T_{1g}$) and eighteen triplet states ($^3T_{1g}$+$^3T_{2g}$ with three spin components for each triplet) summed up. It is clear that the singlet-triplet ISC is significantly slower than for the CASPT2-based QD simulation (see Figure 5 in the main article). The most possible reasons for this discrepancy include the utilized PESs (TD-DFT vs CASPT2) and the exclusion of quintet states in the TSH simulations. We note that this mismatch should not qualitatively affect the dominant normal modes, identified by the TSH simulations. Crucial to this point, we previously found very good agreement between the ground and excited-state (metal-centered) CASPT2 and (TD-)B3LYP* of the $[Fe(1\text{-}H\text{-tetrazole})_6]^{2+}$ complex,[6,24] which is an analogous model with Fe(II)N_6 core. The simulated electronic population and nuclear dynamics (the latter quantified by the normal mode analysis described in the next section) are converged with respect to the number of utilized trajectories (100).

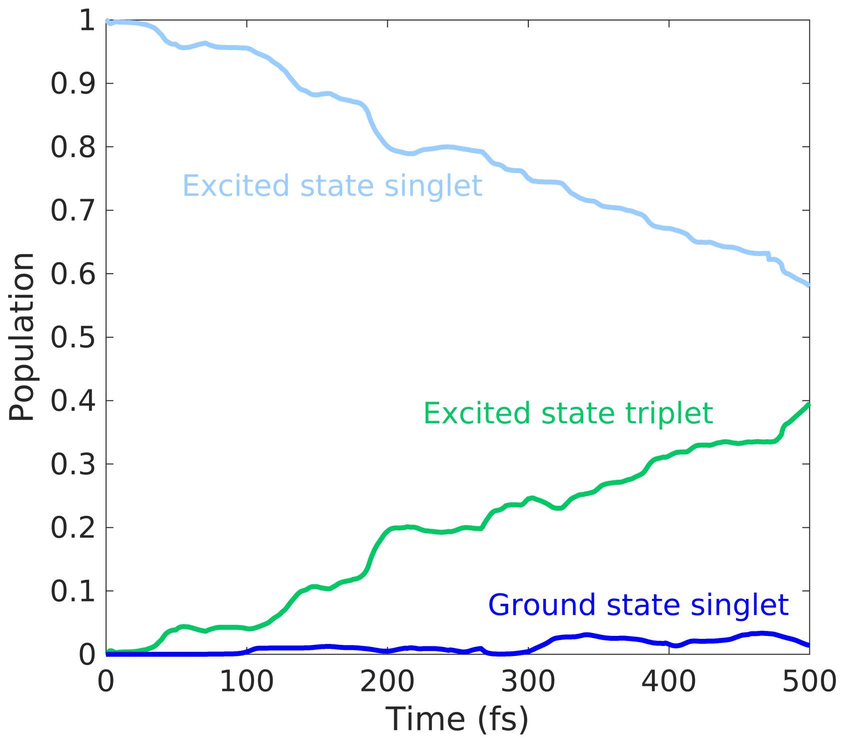

Figure S1: Adiabatic (spin-diabatic) TSH/TD-DFT population dynamics of $[Fe(NCH)_6]^{2+}$, with the singlet and triplet populations summed up.

In order to validate the dominance of the three selected modes ν_{13}, ν_{14}, and ν_{15}, we performed TSH simulations in reduced dimension with Fe-N stretching nuclear motion only and freezing all other nuclear degrees of freedom. This was realized by utilizing only the nuclear gradients along the Fe-N bonds in the dynamics, and constraining the N-C and C-H bond lengths in each NCH ligand using the rattle algorithm.[34] First, we initiated the dynamics from the Franck-Condon (FC) geometry with zero velocities, the adiabatic (spin-diabatic) energies along the trajectory are shown in Figure S2 for the a) full and b) reduced-dimensional case. As is clear from the figure, the two trajectories show excellent agreement, confirming that the excited-state nuclear motion is indeed dictated by the Fe-N stretching modes. There are two differences though that deserve discussion. First, in the full dimensional case, two triplet components form an avoided crossing at ca. 130 fs (Figure S2a), while in reduced dimension, the two triplets only approach each other by 0.15 eV (Figure S2b). Note that this triplet crossing involves Fe-N stretching only (it is not the signature of the involvement of other modes) and does not affect the simulated dynamics, as it occurs at considerably lower energy, during propagation on a singlet surface. Second, and more importantly, in the reduced-dimensional case, the the trajectory enters a triplet PES at 240 fs (see the circles in Figure S2b that represent the energy of the active state), while this does not occur when the nuclear motion is full-dimensional. From this point, the the two trajectories will differ, as they propagate on different surfaces. Note, however, that the difference in singlet-triplet transition is barely a consequence of the stochastic algorithm and the comparison of two single trajectories. To prove this, we carried out reduced-dimensional test TSH simulations for an ensemble of 50 trajectories, for which the initial conditions where generated by a Wigner distribution based on solely the three Fe-N stretching modes, ν_{13}, ν_{14}, and ν_{15}. In Figure S3, we compare the obtained electronic populations to the full-dimensional case, considering the same number of trajectories. The results exhibit a good overall agreement, especially in terms of the dimension reduction from 51D to 3D, validating our simulated TSH dynamics and the three selected dominant modes.

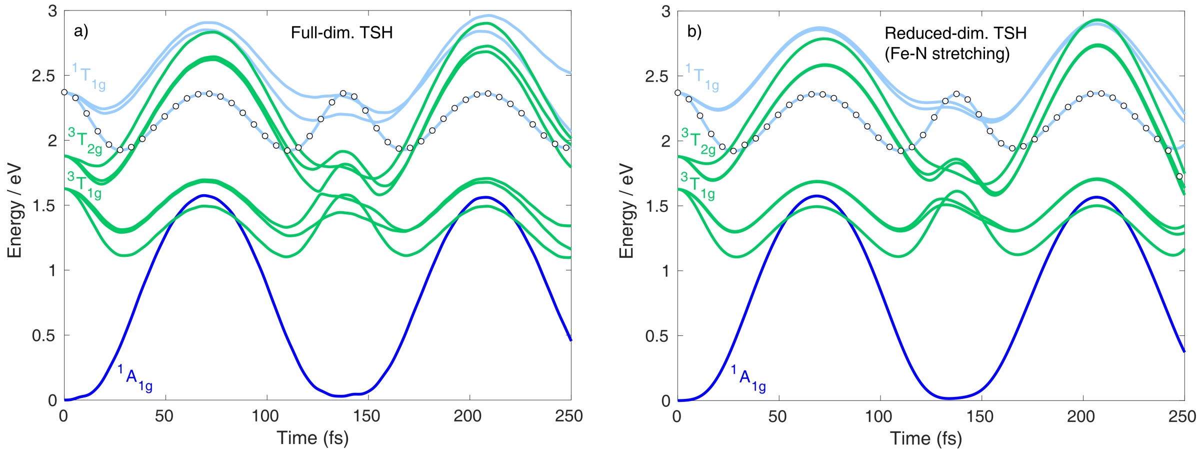

Figure S2: Adiabatic (spin-diabatic) DFT/TD-DFT energies of $[Fe(NCH)_6]^{2+}$ along the trajectory started from the FC geometry with zero velocities for two cases: a) full and b) reduced-dimensional dynamics. For b), nuclear motion only occurs along the Fe-N stretching coordinates. The energies of the active (classically populated) state are indicated by circles. For simplicity, we utilize the electronic state symmetry labels at the FC geometry.

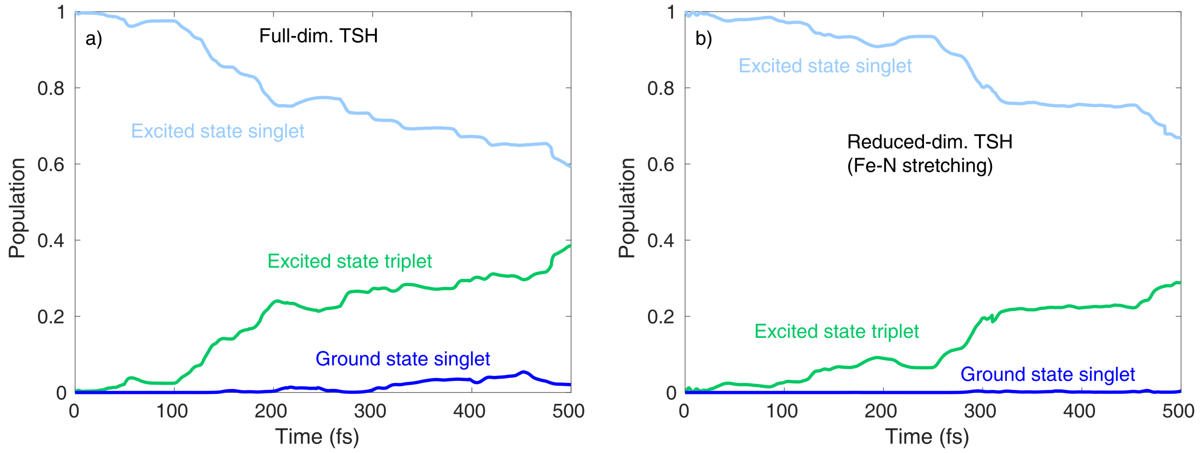

Figure S3: Adiabatic (spin-diabatic) TSH/TD-DFT population dynamics of $[Fe(NCH)_6]^{2+}$ in a) full-D and b) reduced dimension (Fe-N stretching only). The initial conditions were generated using a Wigner distribution based on a) all normal modes and b) the $\nu_{13} - \nu_{15}$ Fe-N stretching modes only.

S3 Normal Mode Analysis

In order to identify the most dominant nuclear motion, we projected the Cartesian displacements relative to the FC geometry on the B3LYP*-calculated ground-state normal modes (Table S1). The dimensionless mass-frequency weighted normal mode coordinates $\boldsymbol{q}(t)$ are obtained by

$$\boldsymbol{q}(t) = \boldsymbol{D}^T \Delta \boldsymbol{r}(t) \ , \tag{S1}$$

where $\Delta \boldsymbol{r}(t)$ are the Cartesian displacements from the trajectories and $\boldsymbol{D}$ is a transformation matrix calculated by diagonalization of the mass-weighted Hessian. The dynamical normal mode activity was calculated by evaluating the standard deviation

$$\sigma_i = \sqrt{\frac{1}{N_{\text{traj}} N_{\text{step}}} \sum_{j=1}^{N_{\text{traj}}} \sum_{k=1}^{N_{\text{step}}} q_{i,j}^2(k\Delta t) - \left(\frac{1}{N_{\text{traj}} N_{\text{step}}} \sum_{j=1}^{N_{\text{traj}}} \sum_{k=1}^{N_{\text{step}}} q_{i,j}(k\Delta t) \right)^2} \ , \tag{S2}$$

which quantifies the total nuclear motion for each normal mode i. Here N_{traj} and N_{step} denotes the number of trajectories (100) and time steps (1001), respectively.

S4 QD Simulations

Quantum dynamics (QD) is the most rigorous approach for the simulation of excited-state dynamics. QD in the converged case delivers the exact solution by solving the time-dependent Schrödinger equation. However, due to exponential scaling, the applicability of exact QD is limited to low/reduced-dimensionality problems (typically < 10 DoF). Nevertheless, QD utilizing reduced-dimensionality model Hamiltonians can deliver invaluable results, provided the modes are well-chosen. In the present work, we utilize the modes selected by the normal mode analysis performed on the TSH trajectories.

For photophysical processes with relatively small amplitude nuclear motion, the vibronic coupling Hamiltonian[35,36] is a very powerful and efficient technique. The method is based on a (quasi-)diabatic electronic basis, in which couplings between electronic states are represented by off-diagonal elements of the electronic Hamiltonian. These potential-like couplings are numerically well-suited for QD. In contrast to the adiabatic basis, in the diabatic representation, kinetic-like couplings, which exhibit singularities at conical intersections, are negligible. This has the important consequence that diabatic states maintain their electronic character along nuclear coordinates. Diabatic states can be constructed by a transformation from the adiabatic basis, termed as diabatization.

The Hamiltonian utilized in our QD simulations has the following form:

$$\boldsymbol{H} = (T_N + V_0)\mathbf{1} + \boldsymbol{W} + \boldsymbol{S} \ . \tag{S3}$$

Here the first two terms define the harmonic oscillator zeroth-order Hamiltonian $\boldsymbol{H}_0 = T_N + V_0$ with the nuclear kinetic energy operator

$$T_N = \frac{1}{2}\sum_i \omega_i \frac{\partial^2}{\partial q_i^2} \tag{S4}$$

and the harmonic ground-state potential

$$V_0 = \frac{1}{2}\sum_i \omega_i q_i^2 \ . \tag{S5}$$

Here q_i is the dimensionless mass-frequency weighted normal coordinate for mode i and $\mathbf{1}$ is a unit matrix with dimension equal to the number of electronic states included in the model. The on-diagonal elements of the $\boldsymbol{W}$ matrix define the diabatic excited-state potentials:

$$W^{(\alpha)} = \varepsilon^{(\alpha)} + \sum_i \kappa_i^{(\alpha)} q_i + \frac{1}{2}\sum_i (\omega_i + \gamma_i^{(\alpha)}) q_i^2 \ . \tag{S6}$$

In Equation S6, $\varepsilon^{(\alpha)}$, $\kappa_i^{(\alpha)}$, and $\gamma_i^{(\alpha)}$ are the zeroth, first, and second-order diagonal expansion coefficients for mode i, state α. The off-diagonal elements of $\boldsymbol{W}$ express the linear nonadiabatic couplings and are given by:

$$W^{(\alpha\beta)} = \sum_i \lambda_i^{(\alpha\beta)} q_i \ . \tag{S7}$$

The $\boldsymbol{S}$ matrix in Equation S3 is the SOC matrix, including singlet-triplet, triplet-triplet, triplet-quintet, and quintet-quintet terms.

The $\kappa_i^{(\alpha)}$, $\lambda_i^{(\alpha\beta)}$, and $\gamma_i^{(\alpha)}$ coefficients were determined by diabatization by ansatz,[15] separately for singlet, triplet, and quintet states. This is based on a fitting procedure, which ensures that the adiabatic potentials resulting from diagonalization of the $\boldsymbol{W}$ potential matrix is in best agreement with those calculated by quantum chemistry.

For the description of electronic structure, we employ CASPT2, which is crucial to access triplet-quintet SOCs (as mentioned, this is not possible using linear response TD-DFT), and at the same time, also providing accurate excited-state energetics.[6,24,37–39] All multiconfigurational self consistent field (CASSCF)/CASPT2 calculations were carried out using the OpenMolcas20.10 program package.[40,41] The selection of the active space in CASSCF is based on previous multiconfigurational works on Fe(II) complexes;[6,24,42] we thus included the two σ-e_g Fe-N bonding orbitals, the three t_{2g} Fe-3d-based orbitals, the two σ^*-e_{g^*} Fe-N antibonding orbitals and the corresponding set of five (three t_{2g} plus two e_{g^*}) Fe-4d-based orbitals, this second shell is important to accurately account for dynamical electron correlation.[43] In addition, we also included the pair of correlating Fe-3s/Fe-4s orbitals, which, similarly as in ref. 4, tend to rotate into the active space. This leads to the active space abbreviated as CAS(12e,14o). In the CASSCF/CASPT2 computations, we froze all core orbitals (Fe-1s, Fe-2s, Fe-2p, N-1s, C-1s) and used and an imaginary level shift of 0.2 a.u., as well as an IPEA shift of 0.25 a.u. for CASPT2.

In order to reduce computational cost, and more importantly, to avoid numerical problems, we apply D_2 point group symmetry in the CASSCF/CASPT2 calculations. The calculations were carried out for one ^{1}A, two $^1B_1$, two $^1B_2$, two $^1B_3$, two $^3B_1$, two two $^1B_2$, two $^1B_3$, one $^5B_1$, one $^5B_2$, and one $^5B_3$ root (these state labels are consistent with D_2 symmetry). The 2-root calculations consist of a state-averaged SA-CASSCF with equal roots and a subsequent multi-state MS-CASPT2 computation. We note that the second root of the singlet $^1B_1/^1B_2/^1B_3$ states, corresponding to the three components of the upper $^1T_{2g}$ manifold, is not included in the dynamics, as their energetically high-lying. However, they are included in the diabatization as some components of the $^1T_{1g}$-$^1T_{2g}$ interact with each other. We utilize the Douglas-Kroll-Hess (DKH) Hamiltonian[44,45] to account for relativistic effects, which is important for the calculation of SOCs, which were calculated at the FC geometry using an approach based on a one-electron effective mean-field SOC Hamiltonian[46] and spin-orbit state interaction (SO-SI).[47,48] We employ an accurate relativistic correlation consistent (ANO-RCC) basis set with the following contractions: $7s6p5d4f3g2h$ for Fe, $4s3p2d1f$ for N, $4s3p1d$ for C, and $2s1p$ for H atoms.

The CASPT2 ground-state minimum along the symmetric Fe-N stretching coordinate is shifted by ca. −0.07 Å from the B3LYP*-optimized geometry. To alleviate this discrepancy, we use the BP86/TZVP-optimized ground-state structure and normal modes (given in Table S1) in the QD simulations, in which case the shift is only ca. −0.03 Å.

In Figures S4–S6, we present the CASPT2-calculated PESs along the three modes (points) and their fit, obtained from diagonalization of the diabatic Hamiltonian. Note that these PESs are termed adiabatic (spin-diabatic), meaning that any geometry the states within a spin multiplicity are energetically ordered at the given geometry, but such energy ordering does not hold for states with different multiplicities. The diabatic potentials along ν_{14} and ν_{15} are shown in Figure 4 of the main article, the diabatic PESs along mode ν_{13} are displayed in Figure S7.

The parameters of the diabatic spin-vibronic Hamiltonian are given below in Tables S2–S11.

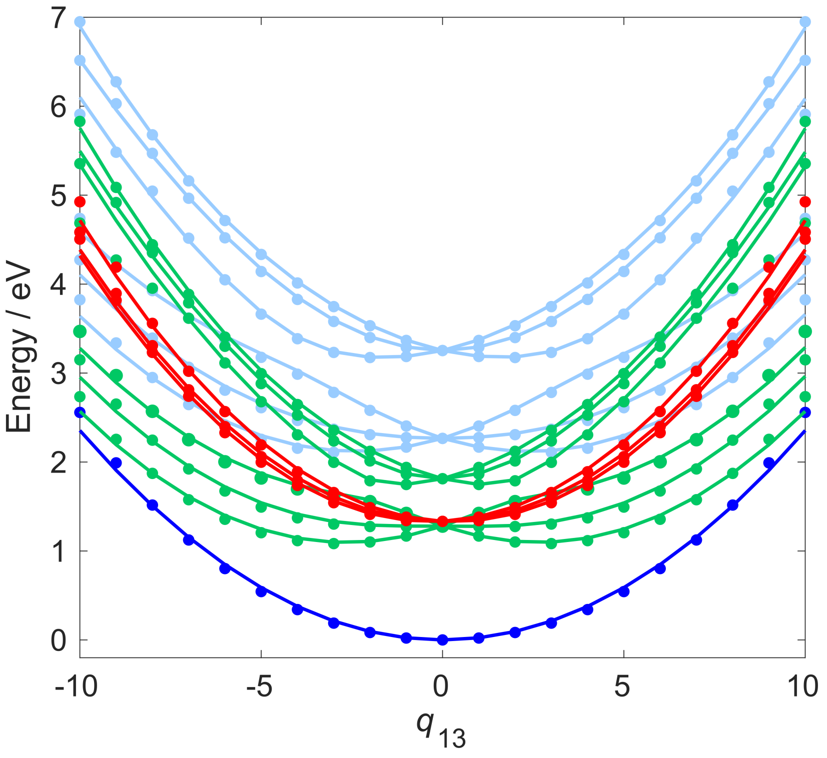

Figure S4: Adiabatic (spin-diabatic) PESs of $[Fe(NCH)_6]^{2+}$ along ν_{13}. The points were calculated by CASPT2, while the lines represent their fit by the diabatic Hamiltonian. Nuclear displacements are given in dimensionless mass-frequency scaled normal coordinates The colours code different spin multiplicities: blue – singlet (ground state – dark blue, excited states – light blue) green – triplet, red – quintet.

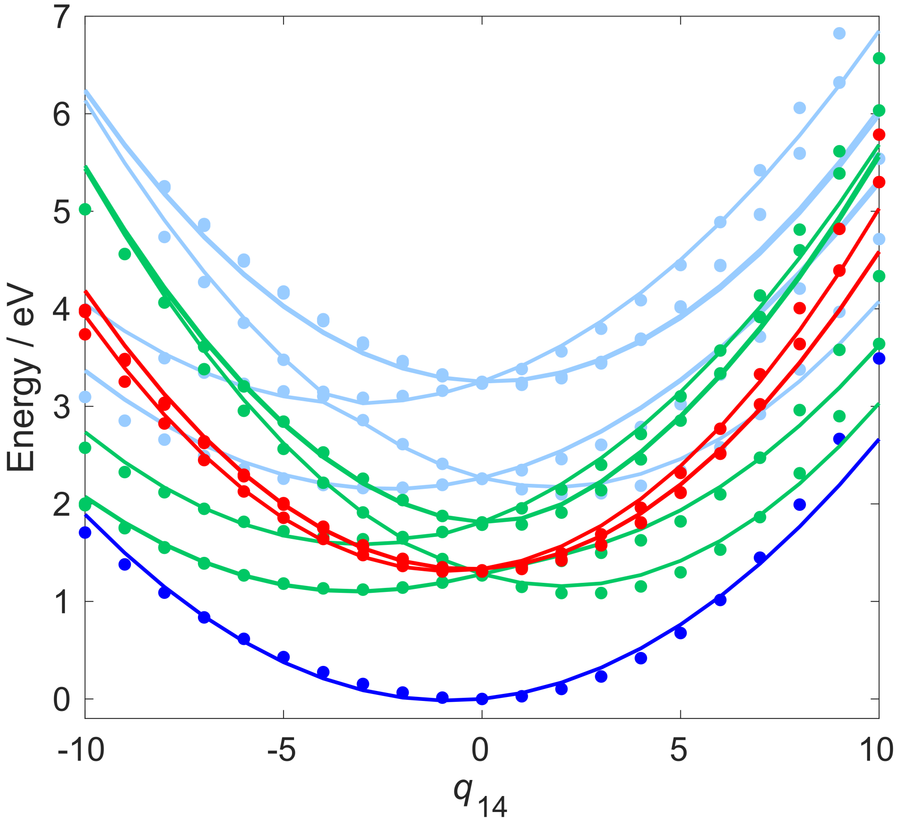

Figure S5: Adiabatic (spin-diabatic) PESs of $[Fe(NCH)_6]^{2+}$ along ν_{14}. The points were calculated by CASPT2, while the lines represent their fit by the diabatic Hamiltonian. Nuclear displacements are given in dimensionless mass-frequency scaled normal coordinates The colours code different spin multiplicities: blue – singlet (ground state – dark blue, excited states – light blue) green – triplet, red – quintet.

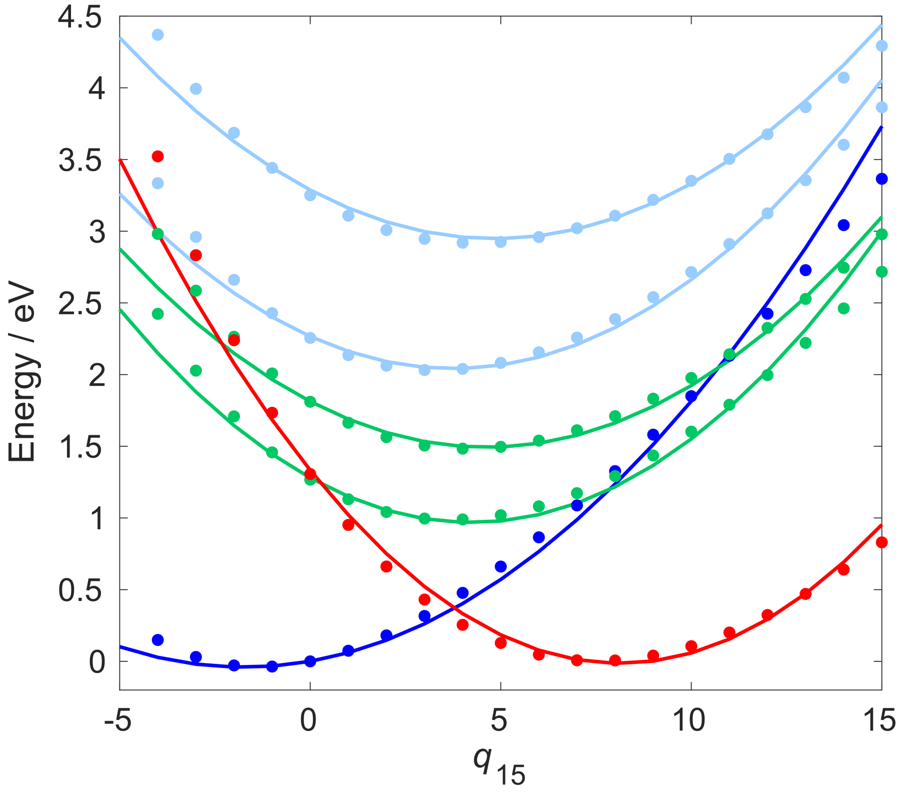

Figure S6: Adiabatic (spin-diabatic) PESs of $[Fe(NCH)_6]^{2+}$ along ν_{15}. The points were calculated by CASPT2, while the lines represent their fit by the diabatic Hamiltonian. Note that for ν_{15}, all off-diagonal coupling elements of the diabatic Hamiltonian are zero, thus the diabatic and adiabatic representations are identical. Nuclear displacements are given in dimensionless mass-frequency scaled normal coordinates. The colours code different spin multiplicities: blue – singlet (ground state – dark blue, excited states – light blue) green – triplet, red – quintet.

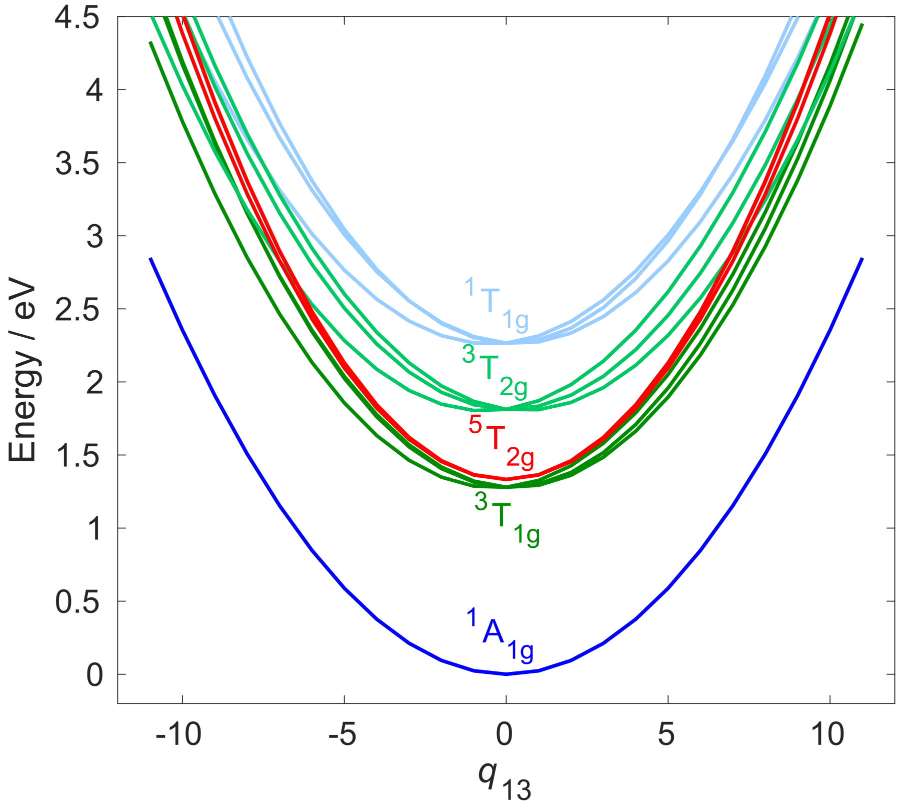

Figure S7: Diabatic CASPT2 PESs of $[Fe(NCH)_6]^{2+}$ along ν_{13}. Nuclear displacements are given in dimensionless mass-frequency scaled normal coordinates. The colours code diabatic states: $^1A_{1g}$ – dark blue, $^1T_{1g}$ – light blue, $^3T_{1g}$ – dark green, $^3T_{2g}$ – light green, $^5T_{2g}$ – red. For simplicity, we keep these octahedral state labels, even if the O_h symmetry is only kept at $q_{13} = 0$.

Table S2: Zeroth-order coefficients $\varepsilon^{(\alpha)}$, given in eV. The tabulated $\varepsilon^{(\alpha)}$ values are the CASPT2-calculated vertical excitation energies at the FC geometry. Both FC adiabatic and diabatic state labels, the latter in parantheses, are given. Note that the S_4–S_6 ($^1T_{2g}$) states were not used in the QD simulations but are reported here for completeness.

State	$\varepsilon^{(\alpha)}$
S_0 (^{1}GS)	0.000
S_1 ($^1T_{1g}$)	2.266
S_2 ($^1T_{1g}$)	2.266
S_3 ($^1T_{1g}$)	2.266
S_4 ($^1T_{2g}$)	3.251
S_5 ($^1T_{2g}$)	3.251
S_6 ($^1T_{2g}$)	3.251
T_1 ($^3T_{1g}$)	1.280
T_2 ($^3T_{1g}$)	1.280
T_3 ($^3T_{1g}$)	1.280
T_4 ($^3T_{2g}$)	1.812
T_5 ($^3T_{2g}$)	1.812
T_6 ($^3T_{2g}$)	1.812
Q_1 ($^5T_{2g}$)	1.333
Q_2 ($^5T_{2g}$)	1.333
Q_3 ($^5T_{2g}$)	1.333

Table S3: First-order diagonal coefficients $\kappa_i^{(\alpha)}$, given in eV. The tabulated $\kappa_i^{(\alpha)}$ values are related to the nuclear gradients at the FC geometry. Both FC adiabatic and diabatic state labels, the latter in parantheses, are given. Note that the S_4–S_6 ($^1T_{2g}$) states were not used in the QD simulations but are reported here for completeness.

State	$\kappa_{13}^{(\alpha)}$	$\kappa_{14}^{(\alpha)}$	$\kappa_{15}^{(\alpha)}$
S_0 (^{1}GS)	0.000	0.039	0.047
S_1 ($^1T_{1g}$)	−0.018	0.096	−0.120
S_2 ($^1T_{1g}$)	−0.008	0.098	−0.120
S_3 ($^1T_{1g}$)	0.024	−0.103	−0.120
S_4 ($^1T_{2g}$)	−0.040	0.140	−0.140
S_5 ($^1T_{2g}$)	0.008	−0.009	−0.140
S_6 ($^1T_{2g}$)	0.033	−0.013	−0.140
T_1 ($^3T_{1g}$)	−0.007	0.096	−0.148
T_2 ($^3T_{1g}$)	0.019	0.095	−0.148
T_3 ($^3T_{1g}$)	−0.014	−0.120	−0.148
T_4 ($^3T_{2g}$)	−0.028	0.147	−0.138
T_5 ($^3T_{2g}$)	−0.005	−0.009	−0.138
T_6 ($^3T_{2g}$)	0.033	−0.013	−0.138
Q_1 ($^5T_{2g}$)	0.000	0.047	−0.332
Q_2 ($^5T_{2g}$)	0.000	0.028	−0.332
Q_3 ($^5T_{2g}$)	0.000	0.020	−0.332

Table S4: Second-order diagonal coefficients $\gamma_i^{(\alpha)}$, given in eV. The tabulated $\gamma_i^{(\alpha)}$ values express the change in vibrational frequencies, relative to DFT-calculated (BP86) grond-state frequencies given in Table S1. Both FC adiabatic and diabatic state labels, the latter in parantheses, are given. Note that the S_4–S_6 ($^1T_{2g}$) states were not used in the QD simulations but are reported here for completeness.

State	$\gamma_{13}^{(\alpha)}$	$\gamma_{14}^{(\alpha)}$	$\gamma_{15}^{(\alpha)}$
S_0 (^{1}GS)	0.006	0.004	−0.017
S_1 ($^1T_{1g}$)	0.011	0.000	−0.012
S_2 ($^1T_{1g}$)	0.018	0.001	−0.012
S_3 ($^1T_{1g}$)	0.008	0.016	−0.012
S_4 ($^1T_{2g}$)	0.005	0.003	−0.015
S_5 ($^1T_{2g}$)	0.010	0.017	−0.015
S_6 ($^1T_{2g}$)	0.006	0.016	−0.015
T_1 ($^3T_{1g}$)	0.016	−0.001	−0.009
T_2 ($^3T_{1g}$)	0.013	−0.001	−0.009
T_3 ($^3T_{1g}$)	0.014	0.018	−0.010
T_4 ($^3T_{2g}$)	0.011	0.001	−0.014
T_5 ($^3T_{2g}$)	0.012	0.024	−0.014
T_6 ($^3T_{2g}$)	0.010	0.024	−0.014
Q_1 ($^5T_{2g}$)	0.020	0.023	−0.003
Q_2 ($^5T_{2g}$)	0.023	0.019	−0.003
Q_3 ($^5T_{2g}$)	0.023	0.020	−0.003

Table S5: First-order off-diagonal coefficients $\lambda_i^{(\alpha\beta)}$, given in eV. The magnitude of the tabulated $\lambda_i^{(\alpha\beta)}$ values characterises the nonadiabatic coupling. Both FC adiabatic and diabatic state labels, the latter in parantheses, are given. Note that the S_4–S_6 ($^1T_{2g}$) states were not used in the QD simulations but are reported here for completeness.

State	$\lambda_{13}^{(\alpha\beta)}$	$\lambda_{14}^{(\alpha\beta)}$	$\lambda_{15}^{(\alpha\beta)}$
S_1 ($^1T_{1g}$) - S_2 ($^1T_{1g}$)	0.099	0.000	0.000
S_1 ($^1T_{1g}$) - S_3 ($^1T_{1g}$)	0.065	0.004	0.000
S_1 ($^1T_{1g}$) - S_4 ($^1T_{2g}$)	−0.002	0.000	0.000
S_1 ($^1T_{1g}$) - S_5 ($^1T_{2g}$)	0.006	0.066	0.000
S_1 ($^1T_{1g}$) - S_6 ($^1T_{2g}$)	0.043	0.021	0.000
S_2 ($^1T_{1g}$) - S_3 ($^1T_{1g}$)	0.015	0.004	0.000
S_2 ($^1T_{1g}$) - S_4 ($^1T_{2g}$)	0.009	0.000	0.000
S_2 ($^1T_{1g}$) - S_5 ($^1T_{2g}$)	0.106	−0.021	0.000
S_2 ($^1T_{1g}$) - S_6 ($^1T_{2g}$)	0.010	0.068	0.000
S_3 ($^1T_{1g}$) - S_4 ($^1T_{2g}$)	−0.056	0.002	0.000
S_3 ($^1T_{1g}$) - S_5 ($^1T_{2g}$)	−0.048	0.001	0.000
S_3 ($^1T_{1g}$) - S_6 ($^1T_{2g}$)	−0.013	0.001	0.000
S_4 ($^1T_{2g}$) - S_5 ($^1T_{2g}$)	0.008	0.001	0.000
S_4 ($^1T_{2g}$) - S_6 ($^1T_{2g}$)	0.067	0.001	0.000
S_5 ($^1T_{2g}$) - S_6 ($^1T_{2g}$)	−0.051	0.000	0.000
T_1 ($^3T_{1g}$) - T_2 ($^3T_{1g}$)	0.024	0.001	0.000
T_1 ($^3T_{1g}$) - T_3 ($^3T_{1g}$)	0.121	0.002	0.000
T_1 ($^3T_{1g}$) - T_4 ($^3T_{2g}$)	0.008	0.001	0.000
T_1 ($^3T_{1g}$) - T_5 ($^3T_{2g}$)	0.078	−0.011	0.000
T_1 ($^3T_{1g}$) - T_6 ($^3T_{2g}$)	0.052	0.091	0.000
T_2 ($^3T_{1g}$) - T_3 ($^3T_{1g}$)	0.050	0.001	0.000
T_2 ($^3T_{1g}$) - T_4 ($^3T_{2g}$)	−0.049	0.001	0.000
T_2 ($^3T_{1g}$) - T_5 ($^3T_{2g}$)	−0.091	0.090	0.000
T_2 ($^3T_{1g}$) - T_6 ($^3T_{2g}$)	0.025	0.010	0.000
T_3 ($^3T_{1g}$) - T_4 ($^3T_{2g}$)	−0.033	0.007	0.000
T_3 ($^3T_{1g}$) - T_5 ($^3T_{2g}$)	0.035	0.000	0.000
T_3 ($^3T_{1g}$) - T_6 ($^3T_{2g}$)	−0.012	0.001	0.000
T_4 ($^3T_{2g}$) - T_5 ($^3T_{2g}$)	0.017	0.000	0.000
T_4 ($^3T_{2g}$) - T_6 ($^3T_{2g}$)	0.091	0.000	0.000
T_5 ($^3T_{2g}$) - T_6 ($^3T_{2g}$)	−0.019	−0.002	0.000
Q_1 ($^5T_{2g}$) - Q_2 ($^5T_{2g}$)	0.002	0.013	0.000
Q_2 ($^5T_{2g}$) - Q_3 ($^5T_{2g}$)	0.020	0.000	0.000

Table S6: CASPT2 singlet-triplet SOC matrix elements (cm^{-1}) calculated at the FC geometry. For simplicity, only the FC adiabatic labels are used, for the corresponding diabatic labels see, e.g., Table S2. Elements not shown in the table are below 1 cm^{-1}.

SOC	$m_s = -1$		$m_s = 0$		$m_s = 1$	
	Re	Im	Re	Im	Re	Im
$\langle S_0 \vert \hat{H}_{SO} \vert T_1 \rangle$	−390.9				−390.9	
$\langle S_0 \vert \hat{H}_{SO} \vert T_2 \rangle$				552.9		
$\langle S_0 \vert \hat{H}_{SO} \vert T_3 \rangle$		−390.9				390.9
$\langle S_1 \vert \hat{H}_{SO} \vert T_2 \rangle$		−64.5				64.5
$\langle S_1 \vert \hat{H}_{SO} \vert T_3 \rangle$				−91.3		
$\langle S_1 \vert \hat{H}_{SO} \vert T_5 \rangle$		173.9				−173.9
$\langle S_1 \vert \hat{H}_{SO} \vert T_6 \rangle$				−245.9		
$\langle S_2 \vert \hat{H}_{SO} \vert T_1 \rangle$		−64.5				64.5
$\langle S_2 \vert \hat{H}_{SO} \vert T_3 \rangle$	64.5				64.5	
$\langle S_2 \vert \hat{H}_{SO} \vert T_4 \rangle$		−173.9				173.9
$\langle S_2 \vert \hat{H}_{SO} \vert T_6 \rangle$	−173.9				−173.9	
$\langle S_3 \vert \hat{H}_{SO} \vert T_1 \rangle$				−91.3		
$\langle S_3 \vert \hat{H}_{SO} \vert T_2 \rangle$	−64.5				−64.5	
$\langle S_3 \vert \hat{H}_{SO} \vert T_4 \rangle$				245.9		
$\langle S_3 \vert \hat{H}_{SO} \vert T_5 \rangle$	−173.9				−173.9	
$\langle S_3 \vert \hat{H}_{SO} \vert T_5 \rangle$	−173.9				−173.9	

Table S7: CASPT2 triplet-triplet SOC matrix elements (cm^{-1}) calculated at the FC geometry. For simplicity, only the FC adiabatic labels are used, for the corresponding diabatic labels see, e.g., Table S2. Elements not shown in the table are below 1 cm^{-1}.

SOC	$m_s = -1$		$m_s = 0$		$m_s = 1$	
	Re	Im	Re	Im	Re	Im
$\langle T_{1,-1}\|\hat{H}_{SO}\| T_2\rangle$				−100.8		
$\langle T_{1,-1}\|\hat{H}_{SO}\| T_3\rangle$		142.5				
$\langle T_{1,-1}\|\hat{H}_{SO}\| T_5\rangle$						
$\langle T_{1,-1}\|\hat{H}_{SO}\| T_6\rangle$		170.6				
$\langle T_{1,0}\|\hat{H}_{SO}\| T_2\rangle$		−100.8				−100.8
$\langle T_{1,0}\|\hat{H}_{SO}\| T_5\rangle$		120.6				120.6
$\langle T_{1,1}\|\hat{H}_{SO}\| T_2\rangle$				−100.8		
$\langle T_{1,1}\|\hat{H}_{SO}\| T_3\rangle$						−142.5
$\langle T_{1,1}\|\hat{H}_{SO}\| T_5\rangle$				120.6		
$\langle T_{1,1}\|\hat{H}_{SO}\| T_6\rangle$						−170.6
$\langle T_{2,-1}\|\hat{H}_{SO}\| T_3\rangle$			100.8			
$\langle T_{2,-1}\|\hat{H}_{SO}\| T_4\rangle$				120.6		
$\langle T_{2,-1}\|\hat{H}_{SO}\| T_6\rangle$			−120.6			
$\langle T_{2,0}\|\hat{H}_{SO}\| T_3\rangle$	−100.8				100.8	
$\langle T_{2,0}\|\hat{H}_{SO}\| T_4\rangle$		120.6				120.6
$\langle T_{2,0}\|\hat{H}_{SO}\| T_6\rangle$	120.6				−120.6	
$\langle T_{2,1}\|\hat{H}_{SO}\| T_3\rangle$			−100.8			
$\langle T_{2,1}\|\hat{H}_{SO}\| T_4\rangle$				120.6		
$\langle T_{2,1}\|\hat{H}_{SO}\| T_6\rangle$			120.6			
$\langle T_{3,-1}\|\hat{H}_{SO}\| T_4\rangle$		170.6				
$\langle T_{3,-1}\|\hat{H}_{SO}\| T_5\rangle$			−120.6			
$\langle T_{3,0}\|\hat{H}_{SO}\| T_5\rangle$	120.6				−120.6	
$\langle T_{3,1}\|\hat{H}_{SO}\| T_4\rangle$						−170.6
$\langle T_{3,1}\|\hat{H}_{SO}\| T_5\rangle$			120.6			
$\langle T_{4,-1}\|\hat{H}_{SO}\| T_5\rangle$				−98.2		
$\langle T_{4,-1}\|\hat{H}_{SO}\| T_6\rangle$		138.9				
$\langle T_{4,0}\|\hat{H}_{SO}\| T_5\rangle$		−98.2				−98.2
$\langle T_{4,1}\|\hat{H}_{SO}\| T_5\rangle$				−98.2		
$\langle T_{4,1}\|\hat{H}_{SO}\| T_6\rangle$						−138.5
$\langle T_{5,-1}\|\hat{H}_{SO}\| T_6\rangle$			98.2			
$\langle T_{5,0}\|\hat{H}_{SO}\| T_6\rangle$	−98.2				98.2	
$\langle T_{5,1}\|\hat{H}_{SO}\| T_6\rangle$			−98.2			

Table S8: CASPT2 triplet-quintet real SOC matrix elements (cm^{-1}) calculated at the FC geometry. For simplicity, only the FC adiabatic labels are used, for the corresponding diabatic labels see, e.g., Table S2. Elements not shown in the table are below 1 cm^{-1}.

Real SOC	$m_s = -2$	$m_s = -1$	$m_s = 0$	$m_s = 1$	$m_s = 2$
$\langle\ T_{2,-1}\vert\hat{H}_{SO}\vert\ Q_3\rangle$	335.4		136.9		
$\langle\ T_{2,0}\vert\hat{H}_{SO}\vert\ Q_3\rangle$		237.1		237.1	
$\langle\ T_{2,1}\vert\hat{H}_{SO}\vert\ Q_3\rangle$			136.9		335.4
$\langle\ T_{3,-1}\vert\hat{H}_{SO}\vert\ Q_2\rangle$	335.4		136.9		
$\langle\ T_{3,0}\vert\hat{H}_{SO}\vert\ Q_2\rangle$		237.1		237.1	
$\langle\ T_{3,1}\vert\hat{H}_{SO}\vert\ Q_2\rangle$			136.9		335.4
$\langle\ T_{5,-1}\vert\hat{H}_{SO}\vert\ Q_3\rangle$	201.5		82.3		
$\langle\ T_{5,0}\vert\hat{H}_{SO}\vert\ Q_3\rangle$		142.5		142.5	
$\langle\ T_{5,1}\vert\hat{H}_{SO}\vert\ Q_3\rangle$			82.3		201.5
$\langle\ T_{6,-1}\vert\hat{H}_{SO}\vert\ Q_2\rangle$	−201.5		−82.3		
$\langle\ T_{6,0}\vert\hat{H}_{SO}\vert\ Q_2\rangle$		−142.5		−142.5	
$\langle\ T_{6,1}\vert\hat{H}_{SO}\vert\ Q_2\rangle$			−82.3		−201.5

Table S9: CASPT2 triplet-quintet imaginary SOC matrix elements (cm^{-1}) calculated at the FC geometry. For simplicity, only the FC adiabatic labels are used, for the corresponding diabatic labels see, e.g., Table S2. Elements not shown in the table are below 1 cm^{-1}.

Imaginary SOC	$m_s = -2$	$m_s = -1$	$m_s = 0$	$m_s = 1$	$m_s = 2$
$\langle T_{1,-1}\|\hat{H}_{SO}\| Q_2\rangle$	335.4		−136.9		
$\langle T_{1,-1}\|\hat{H}_{SO}\| Q_3\rangle$		−335.4			
$\langle T_{1,0}\|\hat{H}_{SO}\| Q_2\rangle$		237.1		−237.1	
$\langle T_{1,0}\|\hat{H}_{SO}\| Q_3\rangle$			−387.3		
$\langle T_{1,1}\|\hat{H}_{SO}\| Q_2\rangle$			136.9		−335.4
$\langle T_{1,1}\|\hat{H}_{SO}\| Q_3\rangle$				−335.4	
$\langle T_{2,-1}\|\hat{H}_{SO}\| Q_1\rangle$	335.4		−136.9		
$\langle T_{2,0}\|\hat{H}_{SO}\| Q_1\rangle$		237.1		−237.1	
$\langle T_{2,1}\|\hat{H}_{SO}\| Q_1\rangle$			136.9		−335.4
$\langle T_{3,-1}\|\hat{H}_{SO}\| Q_1\rangle$		−335.4			
$\langle T_{3,0}\|\hat{H}_{SO}\| Q_1\rangle$			−387.3		
$\langle T_{3,1}\|\hat{H}_{SO}\| Q_1\rangle$				−335.4	
$\langle T_{4,-1}\|\hat{H}_{SO}\| Q_2\rangle$	201.5		−82.3		
$\langle T_{4,-1}\|\hat{H}_{SO}\| Q_3\rangle$		201.5			
$\langle T_{4,0}\|\hat{H}_{SO}\| Q_2\rangle$		142.5		−142.5	
$\langle T_{4,0}\|\hat{H}_{SO}\| Q_3\rangle$			232.7		
$\langle T_{4,1}\|\hat{H}_{SO}\| Q_2\rangle$			82.3		−201.5
$\langle T_{4,1}\|\hat{H}_{SO}\| Q_3\rangle$				201.5	
$\langle T_{5,-1}\|\hat{H}_{SO}\| Q_1\rangle$	−201.5		82.3		
$\langle T_{5,0}\|\hat{H}_{SO}\| Q_1\rangle$		−142.5		142.5	
$\langle T_{5,1}\|\hat{H}_{SO}\| Q_1\rangle$			−82.3		201.5
$\langle T_{6,-1}\|\hat{H}_{SO}\| Q_1\rangle$		−201.5			
$\langle T_{6,0}\|\hat{H}_{SO}\| Q_1\rangle$			−232.7		
$\langle T_{6,1}\|\hat{H}_{SO}\| Q_1\rangle$				−201.5	

Table S10: CASPT2 quintet-quintet real SOC matrix elements (cm^{-1}) calculated at the FC geometry. For simplicity, only the FC adiabatic labels are used, for the corresponding diabatic labels see, e.g., Table S2. Elements not shown in the table are below 1 cm^{-1}.

Real SOC	$m_s = -2$	$m_s = -1$	$m_s = 0$	$m_s = 1$	$m_s = 2$
$\langle Q_{2,-2}\|\hat{H}_{SO}\| Q_3\rangle$		−84.6			
$\langle Q_{2,-1}\|\hat{H}_{SO}\| Q_3\rangle$	84.6		−103.6		
$\langle Q_{2,0}\|\hat{H}_{SO}\| Q_3\rangle$		103.6		−103.6	
$\langle Q_{2,1}\|\hat{H}_{SO}\| Q_3\rangle$			103.6		−84.6
$\langle Q_{2,2}\|\hat{H}_{SO}\| Q_3\rangle$				84.6	

Table S11: CASPT2 quintet-quintet imaginary SOC matrix elements (cm^{-1}) calculated at the FC geometry. For simplicity, only the FC adiabatic labels are used, for the corresponding diabatic labels see, e.g., Table S2. Elements not shown in the table are below 1 cm^{-1}.

Imaginary SOC	$m_s = -2$	$m_s = -1$	$m_s = 0$	$m_s = 1$	$m_s = 2$
$\langle Q_{1,-2}\|\hat{H}_{SO}\| Q_2\rangle$		84.6			
$\langle Q_{1,-2}\|\hat{H}_{SO}\| Q_3\rangle$	−169.2				
$\langle Q_{1,-1}\|\hat{H}_{SO}\| Q_2\rangle$	84.6		103.6		
$\langle Q_{1,-1}\|\hat{H}_{SO}\| Q_3\rangle$		−84.6			
$\langle Q_{1,0}\|\hat{H}_{SO}\| Q_2\rangle$		103.6		103.6	
$\langle Q_{1,1}\|\hat{H}_{SO}\| Q_2\rangle$			103.6		84.6
$\langle Q_{1,1}\|\hat{H}_{SO}\| Q_3\rangle$				84.6	
$\langle Q_{1,2}\|\hat{H}_{SO}\| Q_2\rangle$				84.6	
$\langle Q_{1,2}\|\hat{H}_{SO}\| Q_3\rangle$					169.2

The QD simulations were carried out utilizing the diabatic spin-vibronic Hamiltonian defined above. The time-dependent Schrödinger equation was solved using the multiconfiguration time-dependent Hartree (MCTDH) method as implemented in the Heidelberg MCTDH8.4 program package.[49,50] The MCTDH method is based on a wave function ansatz, constructed by multiconfigurational series of Hartree products of time-dependent one-particle (in the present case, one-mode) basis functions, termed as single particle functions (SPFs). The SPFs are expanded in a time-independent primitive basis. In this work, we employ dimensionless normal mode coordinates and harmonic oscillator eigenfunctions. The size of the utilized SPF and primitive basis sets is given in Table S12:

Table S12: Primitive (N_i) and SPF ($n_i^{(\alpha)}$) bases set sizes used in the QD simulations. The α electronic state index takes the following values: 1: ^{1}GS, $2-4$: $^1T_{1g}$, $5-13$: $^3T_{1g}$, $14-22$: $^3T_{2g}$, $23-37$: $^5T_{2g}$.

Mode	N_i	$n_i^{(\alpha)}$
ν_{13}	81	35,35,35,35,30,30,30,30,30,30,30,30,30,30,30,30,30,30,30,30,30,30,25,25,25,25,30,25,25,25,25,25,25,30,25
ν_{14}	81	35,35,35,35,25,30,30,30,30,30,30,30,30,30,30,30,30,30,30,30,30,30,25,25,25,25,30,25,30,25,25,25,25,25,25
ν_{15}	301	35,35,35,35,25,25,25,25,25,25,25,25,25,25,25,25,25,25,25,25,25,25,25,25,25,25,25,25,25,25,25,25,25,25,25

These basis sets ensure convergence of the dynamics for the 1 ps duration of the QD simulation. The initial wavepacket is built of one-dimensional harmonic oscillator eigenfunctions with zero momentum. The ground-state wavepacket is impulsively excited by projection onto the diabatic PES corresponding to the third component of the $^1T_{1g}$ manifold ($\alpha = 4$). This choice of excitation into a single $^1T_{1g}$ component was made based on our previous work on the accurate description of excitation into electronically doubly-degenerate states.[51]

In order to test the adequacy of the chosen modes in our spin-vibronic model, we have included ν_{16}, a t_{1u} mode with Fe off-center movement and Fe-N-C bending, in our Hamiltonian, which, according to Figure 2, is the highest activity mode not included in our model. Afterwards, we have re-run the QD simulation using this 4D Hamiltonian but no significant change was observed relative to our 3D QD results. This is explained by one order magnitude smaller coupling constants $\lambda_i^{(\alpha\beta)}$ for ν_{16} than for ν_{13} and ν_{14}; the intrastate coefficients $\kappa_i^{(\alpha)}$ for ν_{16} vanish.

S5 DFT-Optimized Ground-State Geometries

Table S13: Cartesian coordinates (Å) of the B3LYP*/TZVP-optimized ground-state geometry of $[Fe(NCH)_6]^{2+}$

Atom	x/Å	y/Å	z/Å
Fe	0.000	0.000	0.000
N	1.961	0.000	0.000
N	−1.961	0.000	0.000
N	0.000	1.961	0.000
N	0.000	−1.961	0.000
N	0.000	0.000	1.961
N	0.000	0.000	−1.961
C	3.106	0.000	0.000
C	−3.106	0.000	0.000
C	0.000	3.106	0.000
C	0.000	−3.106	0.000
C	0.000	0.000	3.106
C	0.000	0.000	−3.106
H	4.181	0.000	0.000
H	−4.181	0.000	0.000
H	0.000	4.181	0.000
H	0.000	−4.181	0.000
H	0.000	0.000	4.181
H	0.000	0.000	−4.181

Table S14: Cartesian coordinates (Å) of the BP86/TZVP-optimized ground-state geometry of $[Fe(NCH)_6]^{2+}$

Atom	x/Å	y/Å	z/Å
Fe	0.000	0.000	0.000
N	1.917	0.000	0.000
N	−1.917	0.000	0.000
N	0.000	1.917	0.000
N	0.000	−1.917	0.000
N	0.000	0.000	1.917
N	0.000	0.000	−1.917
C	3.072	0.000	0.000
C	−3.072	0.000	0.000
C	0.000	3.072	0.000
C	0.000	−3.072	0.000
C	0.000	0.000	3.072
C	0.000	0.000	−3.072
H	4.153	0.000	0.000
H	−4.153	0.000	0.000
H	0.000	4.153	0.000
H	0.000	−4.153	0.000
H	0.000	0.000	4.153
H	0.000	0.000	−4.153

References

[1] Hauser, A. *J. Chem. Phys.* **1991**, *94*, 2741–2748.

[2] Kepenekian, M.; Robert, V.; Le Guennic, B.; De Graaf, C. *J. Comput. Chem.* **2009**, *30*, 2327–2333.

[3] Droghetti, A.; Alfè, D.; Sanvito, S. *J. Chem. Phys.* **2012**, *137*, 124303.

[4] Lawson Daku, L. M.; Aquilante, F.; Robinson, T. W.; Hauser, A. *J. Chem. Theory Comput.* **2012**, *8*, 4216–4231.

[5] Fumanal, M.; Wagner, L. K.; Sanvito, S.; Droghetti, A. *J. Chem. Theory Comput.* **2016**, *12*, 4233–4241.

[6] Ordejón, B.; de Graaf, C.; Sousa, C. *J. Am. Chem. Soc.* **2008**, *130*, 13961–13968.

[7] Sousa, C.; de Graaf, C.; Rudavskyi, A.; Broer, R. *J. Phys. Chem. A* **2017**, *121*, 9720–9727.

[8] Marino, A.; Chakraborty, P.; Servol, M.; Lorenc, M.; Collet, E.; Hauser, A. *Angew. Chem. Int. Ed.* **2014**, *53*, 3863–3867.

[9] Barbatti, M. *WIREs Comput. Mol. Sci.* **2011**, *1*, 620–633.

[10] Crespo-Otero, R.; Barbatti, M. *Chem. Rev.* **2018**, *118*, 7026–7068.

[11] Mai, S.; Marquetand, P.; González, L. *WIREs Comput. Mol. Sci.* **2018**, *8*, e1370.

[12] Capano, G.; Chergui, M.; Rothlisberger, U.; Tavernelli, I.; Penfold, T. J. *J. Phys. Chem. A* **2014**, *118*, 9861–9869.

[13] Eng, J.; Gourlaouen, C.; Gindensperger, E.; Daniel, C. *Acc. Chem. Res.* **2015**, *48*, 809–817.

[14] Pápai, M.; Vankó, G.; Rozgonyi, T.; Penfold, T. J. *J. Phys. Chem. Lett.* **2016**, *7*, 2009–2014.

[15] Penfold, T. J.; Gindensperger, E.; Daniel, C.; Marian, C. M. *Chem. Rev.* **2018**, *118*, 6975–7025.

[16] Mai, S.; Marquetand, P.; González, L. *International Journal of Quantum Chemistry* **2015**, *115*, 1215–1231.

[17] Tully, J. C. *J. Chem. Phys.* **1990**, *93*, 1061–1071.

[18] Plasser, F.; Granucci, G.; Pittner, J.; Barbatti, M.; Persico, M.; Lischka, H. *J. Chem. Phys.* **2012**, *137*, 22A514.

[19] Granucci, G.; Persico, M.; Zoccante, A. *J. Chem. Phys.* **2010**, *133*, 134111.

[20] Neese, F. *WIREs Comput. Mol. Sci.* **2012**, *2*, 73–78.

[21] Neese, F. *WIREs Comput. Mol. Sci.* **2018**, *8*, e1327.

[22] Reiher, M.; Salomon, O.; Artur Hess, B. *Theor. Chem. Acc.* **2001**, *107*, 48–55.

[23] Reiher, M. *Inorg. Chem.* **2002**, *41*, 6928–6935.

[24] Pápai, M.; Vankó, G.; de Graaf, C.; Rozgonyi, T. *J. Chem. Theory Comput.* **2013**, *9*, 509–519.

[25] Saureu, S.; de Graaf, C. *Phys. Chem. Chem. Phys.* **2016**, *18*, 1233–1244.

[26] Leshchev, D.; Harlang, T. C. B.; Fredin, L. A.; Khakhulin, D.; Liu, Y.; Biasin, E.; Laursen, M. G.; Newby, G. E.; Haldrup, K.; Nielsen, M.; Wärnmark, K.; Sundström, V.; Persson, P.; Kjær, K. S.; Wulff, M. *Chem. Sci.* **2018**, *9*, 405–414.

[27] Sárosiné Szemes, D.; Keszthelyi, T.; Papp, M.; Varga, L.; Vankó, G. *Chem. Commun.* **2020**, *56*, 11831–11834.

[28] Hirata, S.; Head-Gordon, M. *Chem. Phys. Lett.* **1999**, *314*, 291 – 299.

[29] Neese, F.; Wennmohs, F.; Hansen, A.; Becker, U. *Chem. Phys.* **2009**, *356*, 98–109, Moving Frontiers in Quantum Chemistry:.

[30] de Souza, B.; Farias, G.; Neese, F.; Izsák, R. *J. Chem. Theory Comput.* **2019**, *15*, 1896–1904, PMID: 30721046.

[31] Neese, F. *J. Chem. Phys.* **2005**, *122*, 034107.

[32] Lenthe, E. v.; Baerends, E. J.; Snijders, J. G. *J. Chem. Phys.* **1993**, *99*, 4597–4610.

[33] van Wüllen, C. *J. Chem. Phys.* **1998**, *109*, 392–399.

[34] Andersen, H. C. *J. Comput. Phys.* **1983**, *52*, 24–34.

[35] Cederbaum, L. S.; Köppel, H.; Domcke, W. *Int. J. Quantum Chem.* **1981**, *20*, 251–267.

[36] Köppel, H.; Domcke, W.; Cederbaum, L. S. *Advances in Chemical Physics*; John Wiley & Sons, Ltd, 1984; pp 59–246.

[37] de Graaf, C.; Sousa, C. *Chem. Eur. J.* **2010**, *16*, 4550–4556.

[38] de Graaf, C.; Sousa, C. *Int. J. Quantum Chem.* **2011**, *111*, 3385–3393.

[39] Sousa, C.; de Graaf, C.; Rudavskyi, A.; Broer, R.; Tatchen, J.; Etinski, M.; Marian, C. M. *Chem. Eur. J.* **2013**, *19*, 17541–17551.

[40] Fdez. Galván, I. et al. *J. Chem. Theory Comput.* **2019**, *15*, 5925–5964.

[41] Aquilante, F. et al. *J. Chem. Phys.* **2020**, *152*, 214117.

[42] Pierloot, K.; Vancoillie, S. *J. Chem. Phys.* **2008**,

[43] Andersson, K.; Roos, B. O. *Chem. Phys. Lett.* **1992**, *191*, 507–514.

[44] Douglas, M.; Kroll, N. M. *Ann. Phys.* **1974**, *82*, 89–155.

[45] Hess, B. A. *Phys. Rev. A* **1986**, *33*, 3742–3748.

[46] Heß, B. A.; Marian, C. M.; Wahlgren, U.; Gropen, O. *Chem. Phys. Lett.* **1996**, *251*, 365–371.

[47] Malmqvist, P. Å.; Roos, B. O.; Schimmelpfennig, B. *Chem. Phys. Lett.* **2002**, *357*, 230–240.

[48] Roos, B. O.; Malmqvist, P.-Å. *Phys. Chem. Chem. Phys.* **2004**, *6*, 2919–2927.

[49] Meyer, H.-D.; Manthe, U.; Cederbaum, L. *Chem. Phys. Lett.* **1990**, *165*, 73 – 78.

[50] Beck, M.; Jäckle, A.; Worth, G.; Meyer, H.-D. *Phys. Rep.* **2000**, *324*, 1 – 105.

[51] Pápai, M.; Simmermacher, M.; Penfold, T. J.; Møller, K. B.; Rozgonyi, T. *J. Chem. Theory Comput.* **2018**, *14*, 3967–3974.
